# *q*-Diffusion leverages the full dimensionality of gene coexpression in single-cell transcriptomics

**DOI:** 10.1038/s42003-024-06104-w

**Published:** 2024-04-02

**Authors:** Myrl G. Marmarelis, Russell Littman, Francesca Battaglin, Donna Niedzwiecki, Alan Venook, Jose-Luis Ambite, Aram Galstyan, Heinz-Josef Lenz, Greg Ver Steeg

**Affiliations:** 1 Information Sciences Institute, University of Southern California, 4676 Admiralty Way, Marina del Rey, CA 90292 USA; 2 University of California Los Angeles, Los Angeles, CA 90095 USA; 3 Keck School of Medicine, University of Southern California, 1975 Zonal Ave., Los Angeles, CA 90033 USA; 4 Duke University, Durham, NC 27708 USA; 5 University of California San Francisco, San Francisco, CA 94143 USA; 6 University of California Riverside, Riverside, CA 92521 USA

**Keywords:** Machine learning, Statistical methods, Predictive markers, Transcriptomics, Predictive medicine

## Abstract

Unlocking the full dimensionality of single-cell RNA sequencing data (scRNAseq) is the next frontier to a richer, fuller understanding of cell biology. We introduce *q*-*diffusion*, a framework for capturing the coexpression structure of an entire library of genes, improving on state-of-the-art analysis tools. The method is demonstrated via three case studies. In the first, *q*-diffusion helps gain statistical significance for differential effects on patient outcomes when analyzing the CALGB/SWOG 80405 randomized phase III clinical trial, suggesting precision guidance for the treatment of metastatic colorectal cancer. Secondly, *q*-diffusion is benchmarked against existing scRNAseq classification methods using an in vitro PBMC dataset, in which the proposed method discriminates IFN-*γ* stimulation more accurately. The same case study demonstrates improvements in unsupervised cell clustering with the recent Tabula Sapiens human atlas. Finally, a local distributional segmentation approach for spatial scRNAseq, driven by *q*-diffusion, yields interpretable structures of human cortical tissue.

## Abstract (web summary)

The q-diffusion method uses the full dimensionality of gene coexpression in transcriptomics and benchmarking shows improvment of scRNAseq analyses.

## Introduction

A cell’s phenotype is determined largely by the proteins that it expresses. Though progress has been made on directly measuring proteins in single cells^1^ (via proteomics,) the full proteome is still an unwieldy proposition^2^ due to the vast diversity in protein shapes and their chemical properties. Thankfully, RNA transcripts correspond to proteins currently under production and offer important insights into cellular phenotypes. Modern advancements in single-cell RNA sequencing (scRNAseq) have led to consistent decreases in cost, enabling the characterization of complex biological processes—even with spatial resolution^3^.

However, few computational methods exist to study the large combinatorial interactions between genes that form biological processes, which new scRNAseq datasets promise to capture^4^. The discrepancy between acquisition and analysis is palpable in the algorithms themselves: toolkits in the state of the art rely on combinations of aggressive feature selection^5,6^, dimensionality reduction^7–9^, or marker-gene identification^10^. Each of these stages in the analysis pipeline discards a majority of the potential gene interactions available in the original scRNAseq data. Those steps are not without good reason: scRNAseq analysis suffers from the curse of dimensionality, where the number of genes is too great to study all of them together, even in the relatively large samples that are now feasible. Compounding this issue is the noise, both technical and physiological, exacerbated by the granularity of scRNAseq.

The *q*-diffusion method presented in this paper enables scRNAseq analysis to extract higher-order structures from the data that other methods cannot. The general method hinges on a core novelty: a geometry of cells in transcriptomic observational space. Concretely, a *q*-*diffused kernel* function characterizes the transcriptional proximity of any two cells. This kernel supports arbitrary dimensionality, thus overcoming the “curse of dimensionality.” Notably it exhibits a multiscale nature that reveals biological processes (noisily) diffused across many genes. Internally, the kernel accounts for interactions of high order by incorporating not only pairwise (bilinear) terms, but all possible combinations (trilinear, quadrilinear, …) of variables in the data. These additional terms effectively shift the focus of the kernel to large-scale, possibly low-magnitude interactions of gene activities, as opposed to considering each gene’s activity on its own. The additional benefits conferred by this *q*-diffused geometry include robustness to noise and sample efficiency.

This paper ventures into three separate applications. The first demonstrates the propensity of *q*-diffusion for revealing biologically informative structure. We base that judgment on medical relevance, deemed through predictive capacity on downstream clinical outcomes in a phase III clinical trial. These results suggest precision guidance for the treatment of metastatic colorectal cancer (mCRC). The second case study for *q*-diffusion is benchmarked against the most popular scRNAseq clustering methods to discriminate IFN-*γ* stimulation in eight peripheral blood mononuclear cell (PBMC) subtypes more accurately. An additional benchmark assesses the unsupervised clustering of non-PBMC cells in small tissue samples from four human organs. Finally, the *q*-diffused framework is harnessed in developing an unsupervised local distributional segmentation (LDS) technique to segment structural regions of the human cerebral cortex.

## Results

Common tasks in scRNAseq analysis include clustering, factorizing, and classifying the cells. Respectively, these entail grouping cells based on a notion of similarity, identifying common components among cells such as gene expression programs (GEPs), and assigning phenotypic labels to cells. All such tasks benefit from, or even require a way to quantify the relation of one cell to another with regards to their gene expressions. With estimating GEPs, a helpful statistical regularization would be to favor programs that are expressed in cells that are similar to each other overall. Under *q*-diffusion, cell-to-cell similarity is quantified by a kernel-like function that automatically uses all orders of interaction to quantify the magnitude of a (properly scaled; see Method for details) vector *v* of gene-expression differences:

$$\underbrace{v_1^2 + v_2^2 + v_3^2 + \ldots}_{{{{{{{\mathrm{first}}}}}}\,{{{{{\mathrm{order}}}}}}\,({{{{{\mathrm{Euclidean}}}}}})}} \, + \, \underbrace{\alpha(v_1^2 v_2^2 + v_1^2 v_3^2 + v_2^2 v_3^2 + \ldots)}_{{{{{{{\mathrm{second}}}}}}\,{{{{{\mathrm{order}}}}}}\,({{{{{\mathrm{pairs}}}}}})}}\ +\ \underbrace{\alpha^2(v_1^2 v_2^2 v_3^2 + \ldots)}_{{{{{{{\mathrm{third}}}}}}\,{{{{{\mathrm{order}}}}}}}} \\ + \underbrace{\alpha^3(\cdots) + \alpha^4(\cdots) + \ldots}_{{{{{{{\mathrm{higher}}}}}}\,{{{{{\mathrm{order}}}}}}}} \quad {{{\mbox{with}}}}\,\, 0 \, < \, \alpha \, < \, 1.$$

**Fig. 1:**
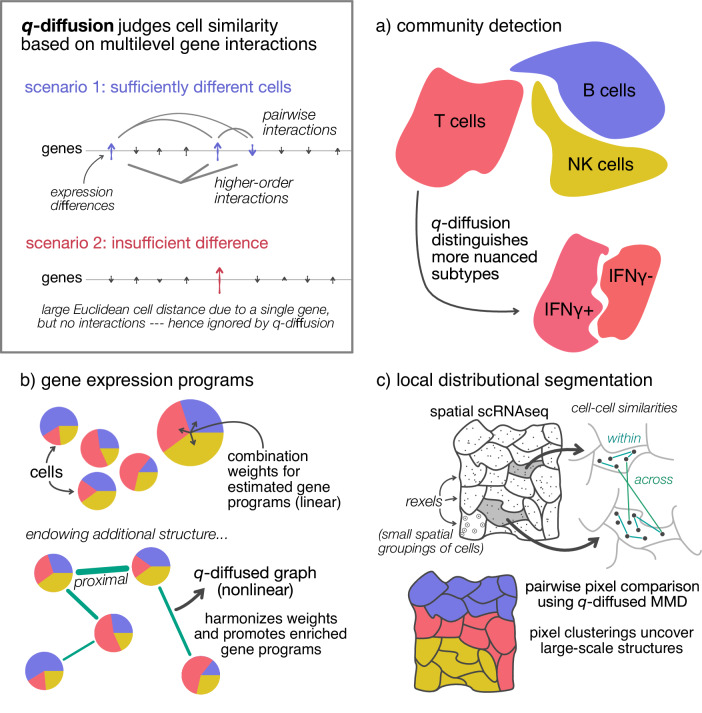
Schematic of the mechanisms behind *q*-diffusion. When comparing two cells, the kernel fundamentally values expression differences that occur in many genes concurrently. It can enter and augment several common analyses: **a** *q*-Diffusion facilitates nuanced phenotype resolution via community detection, as with the second case study in this paper. **b** *q*-Diffusion can regularize gene expression program (GEP) estimators like nonnegative matrix factorization (NMF), to promote statistical enrichment of gene ontologies (first and third case studies). **c** Recent spatial scRNAseq modalities present a new opportunity for macro-segmentation based on cellular transcriptomics, like in the brain (third case study). We present a local distributional segmentation (LDS) algorithm that relies on *q*-diffusion applied to maximum mean discrepancy (MMD), an established kernel-based statistic.

For all *q*-diffused tasks described in this paper, the *q*-diffused kernel was evaluated between all pairs of cells, and then the adjacency matrix was symmetrified to produce a weighted, undirected graph of the cells. This graph supplemented downstream analysis in the manners summarized by Fig. 1. Namely, performing community detection directly produced cell clusters. Indirectly, the graph was also used to further constrain the factorization of cells into expression programs, lowering the risk of underdetermination. More applications are described later.

Three diverse case studies are showcased on *q*-diffusion applied to human scRNAseq data. Each case enhances the findings of the original analyses by exploiting the full transcriptome.

### First case study on treatment of colon cancer

We investigated whether the full dimensionality of the transcriptome from the tumor microenvironment of metastatic colorectal cancer (mCRC) could reveal novel treatment opportunities. mCRC is extremely heterogenous not only from patient to patient but also between metastatic sites or even within a single location^11,12^. In order to convincingly validate the biological and medical utility of *q*-diffusion, this section reports on whether the *q*-diffused structure discovered in an scRNAseq mCRC atlas could produce novel insights on existing records from a large clinical trial.

We took two distinct approaches for discovering the *q*-diffused structure in a transferable representation. Both were unsupervised statistical estimators of latent variables in the transcriptome. We developed them as *q*-diffused counterparts to well-established techniques. These two approaches differed in objective. The first sought to represent cells in a small linear (nonnegative) basis that can be interpreted as gene expression programs (GEPs), via a *q*-diffused form of nonnegative matrix factorization (NMF) that we term *q*NMF. These GEPs, which are supposed to capture common biological processes or groups thereof, could easily be transferred to other datasets by projecting new expression profiles onto them. The second approach aimed to map the cells in a low-dimensional Euclidean space to make phenotypic relations apparent. A number of embedding methods exist to construct this mapping, of which PHATE^13^ is celebrated for its reliability in describing complex biological structures^14^. PHATE with a *q*-diffused kernel yielded a new kind of full-transcriptome embedding that we term *q*PHATE. In both avenues of investigation, we compared the *q*-diffused results to their analogs produced by standard methods without *q*-diffused augmentation.

The *q*NMF and *q*PHATE representations of discovered *q*-diffused structure were validated for biological and medical utility by testing them out of sample, since that is the gold standard in machine learning. The procedure focused on downstream clinical relevance. We hypothesized, teleologically, that the transfer of discovered structures onto new patients in a clinical trial would facilitate strong statistical predictions of clinical outcomes only if those structures were useful and biologically coherent. We emphasize that the *q*-diffused structures were discovered without supervision, prior to the incorporation of any outcome or treatment information from the clinical trial.

#### The data

The Human Colon Cancer Atlas (c295)^15^ that includes malignant and infiltrating immune cells served as a reference scRNAseq dataset with 26,980 genes across the 17,362 cells matching in disease condition (stage 4) to the bulk RNA from the clinical trial. The latter was the Cancer and Leukemia Group B (CALGB)/Southwest Oncology Group (SWOG) 80,405 randomized phase III trial in first-line mCRC patients treated with bevacizumab, cetuximab, or both, plus chemotherapy^16,17^. The first two treatment arms are considered standard of care for newly diagnosed mCRC. To discern drug-specific effects we sought differential outcomes between the treatment arms. The trial had bulk RNA profiles from 557 patients with 56,674 genes. The allocation of bevacizumab/cetuximab/both treatments was 227/207/123, with the third arm having been discontinued early. Bevacizumab and cetuximab are abbreviated as bev and cet, respectively. CALGB is now part of the Alliance for Clinical Trials in Oncology.

#### The statistical evaluation

**Fig. 2:**
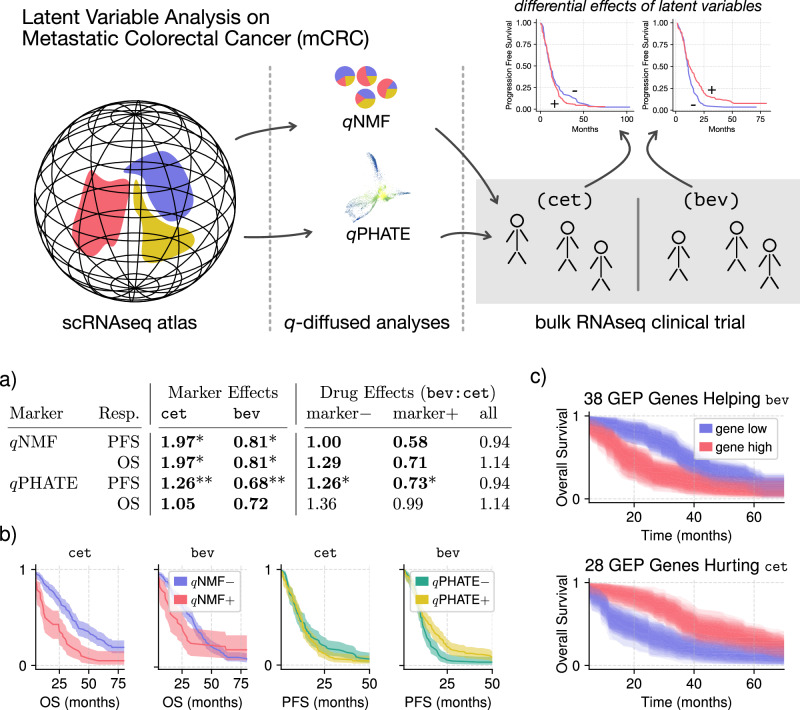
Schematic of the methodology for estimating latent variables in the scRNAseq atlas and then deconvolving them into the clinical-trial patient sample in order to assess their potential as biomarkers that inform clinical outcomes. The two outcomes investigated were progression-free survival (PFS) and overall survival (OS) in accordance with the clinical trial’s protocol. Differential effects were measured by heterogeneity of multivariate Cox proportional hazards. **a** Hazard-ratio point estimates for patient biomarkers. Under “Marker Effects,” we compare hazard ratios of biomarkers between cet and bev groups. Under “Drug Effects,” we compare hazard ratios of bev to cet between biomarker groups. We test for significant differential effects between groups. In contrast with the *q*-diffusion results listed here, the structures uncovered by classical NMF and PHATE failed to produce biomarkers with any significant differential effects. **Bold**: FDR < 0.1; Bold*: FDR < 0.05; Bold**: FDR < 0.01. **b** Kaplan–Meier estimates of survivals with 95% confidence illustrating the identified differential marker effects under the two treatments. **c** The *q*NMF biomarker appears to help bev overall survival (OS) and hurt cet according to **a**. A number of member genes in the GEP were individually associated with these differential outcomes, as determined by *U*-tests with FDR < 0.01. Survivals (90% confidence) are stratified by upper and lower quartiles of expression.

First we assessed whether the drug (cet or bev) acted as an effect modifier^18,19^ on the biomarker (*q*NMF or *q*PHATE) for clinical outcomes. Conversely, we looked at the biomarker as an effect modifier on the drug. In either case we tested for differential effects of one binary variable between strata of the other binary variable. As mentioned in Fig. 2, we estimated multivariate Cox proportional hazards for progression-free survival (PFS) and overall survival (OS). The regressions included as covariates the type of chemotherapy, tumor side^20^, sex, age, Eastern Cooperative Oncology Group (ECOG) performance score^21^, and common tumor mutations. We excluded the minority of patients with high microsatellite instability (MSI-H)^22^, who generally require different protocols altogether.

**Fig. 3:**
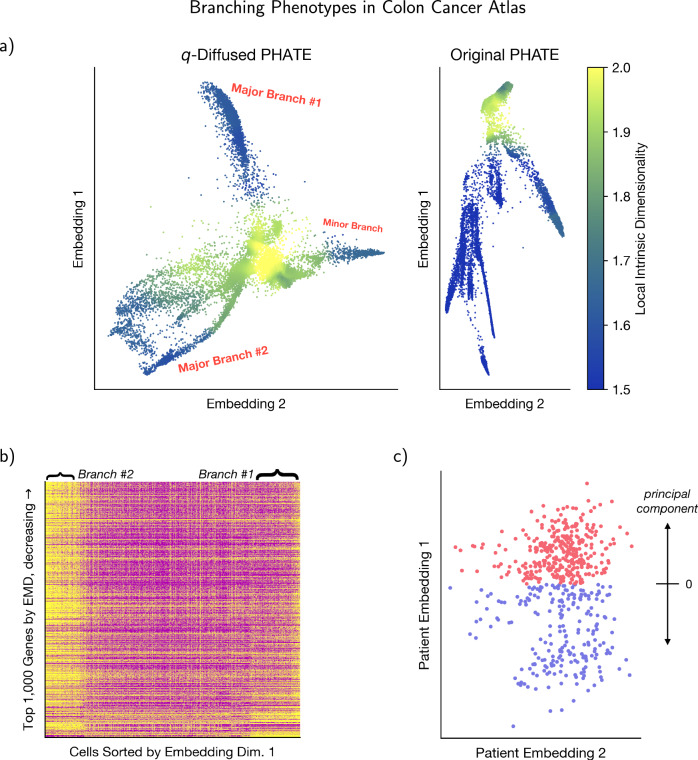
Exploration of *q*PHATE and its branched genes revealed in the mCRC scRNAseq atlas. **a** *q*-Diffused and original PHATE embeddings of the atlas cells. Plots are sized to their true aspect ratios. Cells are colored by their estimated local intrinsic dimensionality (LID), which highlights possible branching points. Branches are annotated in red. They are less clear in the original PHATE embedding. See Supplementary Results for a rigorous investigation. Major branch #1 had about ~19% of the cells, major branch #2 about ~9% of the cells, and the minor branch accounted for ~4% of the cells. Supplementary Fig. 3 provides details and further evidence for our approach on defining branches in the embedding. **b** Major branches #1& 2 were contrasted against each other to screen for genes that appear to drive the branching. Expressions of the top screened genes are displayed in this heatmap. **c** The scRNAseq embedding in **a** was translated to the clinical-trial patients by linear projection. Then the principal component of that embedding was discretized around its mean for downstream analysis as a putative biomarker.

In prior work, GEPs were estimated in the atlas by means of NMF^15^, as is established practice^23,24^. In this work we estimated *q*NMF and NMF GEPs and contrasted their affinity for differential effects. Cox regressions were performed on the nonnegative GEP weights to first identify the GEP with a significant differential effect at false discovery rate (FDR) below 0.05. We observed that *q*NMF produced one such GEP and NMF produced none. We then binarized the patients’ weights for that GEP based on sparsity: 0 for zero and 1 for nonzero, obtaining the *q*NMF biomarker. Analogously for *q*PHATE, the patients were projected to the scRNAseq latent space and then binarized, as detailed in Fig. 3. These biomarkers allowed the production of the result table in Fig. 2a. The strongly identified effects were also plotted as survivals^25^ in Fig. 2b and the GEP genes individually associated with outcomes were further characterized in Fig. 2c. Most notably for our proposal of *q*-diffusion, classical NMF or PHATE biomarkers were not informative enough on clinical outcomes to produce a comparable Fig. 2a with statistically significant differential effects in either setting—drug effects or marker effects.

The *q*PHATE embedding coordinates transferred onto patients were thresholded along their principal component, shown in Fig. 3c, for a simple binary biomarker that could be tested for hazards. We remark that clinical outcome-related findings were robust to this discretization procedure. Bootstrap resampling of the patients revealed that the strong differential hazard on PFS shown in Figure 2a remains strong (*p* < 0.05) for 95% of the simulated (projected, then thresholded) samples. On interpreting the major scRNAseq branches of Fig. 3a, we note that they differ in 197/204 (97%) of the original GEPs^15^, with *U*-test FDR < 10^−3^, suggesting modulation of the whole tumor microenvironment.

### Second case study on clustering phenotypes

#### Distinguishing complex cell conditions

We sought to study the ability of the *q*-diffused kernel to discern phenotypes that are spread across many genes. PBMCs are common in scRNAseq benchmarks^7,10,26^ due to their well-understood subpopulations. They are also studied often because they are involved in circulation, and are entangled with many diseases. We obtained an existing PBMC dataset^27^ where a single batch contained cells from the same lineage, under two different but known conditions. The single-batch multiplexing^28^ avoids the problem of disentangling significant batch-related noise from actual differences in cell conditions. The two conditions studied were stimulation and non-stimulation (control) by interferom gamma (IFN-*γ*), a cytokine known to induce complex changes in PBMCs through signaling pathways^7^. IFN-*γ* is involved in many distinct immune-related processes^29^, and would be expected to modulate many groups of genes. For this reason, we postulated that *q*-diffusion would help describe IFN-*γ* stimulation across the different PBMCs, in contrast with other methods that rely on reduced dimensionality. The dataset contained 14,039 cells with a gene library of size 17,796. The cell population contained 7466 control and 6573 stimulated cells.

**Fig. 4:**
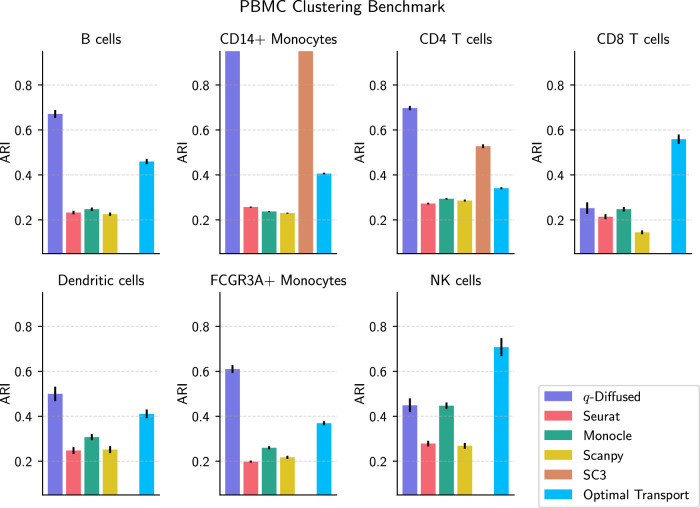
Adjusted Rand indices (ARIs)^31^ of Leiden community detection^42^ in the PBMC dataset. Bars represent the alignment of clusters with IFN-*γ* stimulation and control conditions for each of the cell types. Error bars are computed from bootstrapped estimator standard deviations. Our *q*-diffused kernel-induced graph structure is compared to the neighborhood graph methods in Seurat^7^, Monocle^8^, Scanpy^9^, and optimal transport [e.g. ref. 50]. Clusters computed by SC3^85^ are included as well.

Comparisons in Fig. 4 assessed each method’s ability to distinguish stimulated and control PBMCs. They were scored on the basis of the adjusted Rand index (ARI)^30,31^ between detected communities and the true conditions. The ARI is a standard metric for assessing the quality of unsupervised clustering methods^26^, by measuring coherence/alignment with ground-truth labels. Performance for megakaryocytes was not displayed because all scores were essentially zero. In most cases, the *q*-diffused graph structure outperformed the others. Notable exceptions include CD8 T cells, for which the existing methods performed better, and natural killer (NK) cells, for which performance was similar under a few methods.

#### Sample-efficient organ tissue classification

The Tabula Sapiens Consortium^32^ recently sequenced the single-cell transcriptomes of multiple human organs, and manually annotated the individual phenotypes. This atlas promises to facilitate understanding of intercellular dynamics across the human body^33^. Methods to cluster cells by phenotype are a crucial tool in the annotation pipeline. As the Tabula Sapiens annotations were verified manually by domain experts, they presented a benchmark for clustering that was fair, in that the annotations were putatively less biased towards the established clustering methods. To highlight the sample efficiency of *q*-diffusion, we selected those organ tissues for which fewer than 10,000 cells were sampled. We also screened out the PBMCs in order to focus on organ-specific phenotypes—also because PBMCs are already well characterized by existing tools, as discussed in the second case study.

**Fig. 5:**
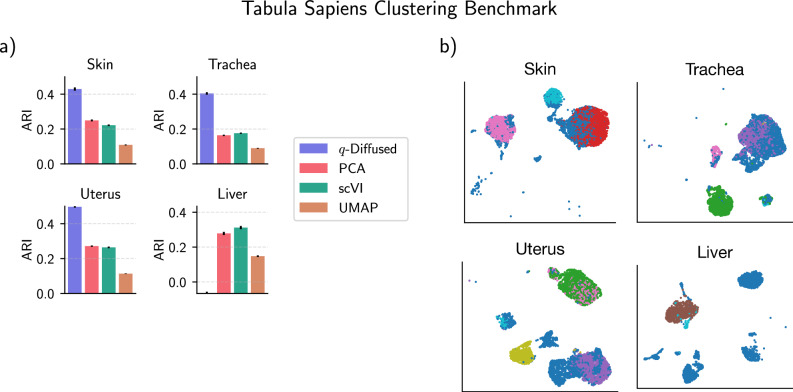
The benchmark involving the Tabula Sapiens^32^ human atlas. **a** ARIs of Leiden community detection with *q*-diffusion versus the state of the art in dimensionality reduction for Tabula Sapiens. Clusters were scored against the expert-assisted annotations in the four sans-PBMC small-sample organs. **b** UMAP embeddings of the organ tissues colored by the unsupervised *q*-diffusion clusters.

The Tabula Sapiens datasets were packaged with state-of-the-art dimensionality reductions, including scVI^34^. We elected to compare *q*-diffusion directly head-to-head against those embeddings, which were assumed to be optimized for their respective datasets. In Fig. 5, *q*-diffusion shows improved clustering alignment with the manual annotations, in contrast with the other embeddings, for three of the four datasets. These organs were the skin (4918 cells kept out of 9424), trachea (6894 cells keps out of 9522), and uterus (6154 cells kept out of 7124), with the exception of the liver (2506 cells kept out of 5007) exhibiting degraded clusters from *q*-diffusion.

**Fig. 6:**
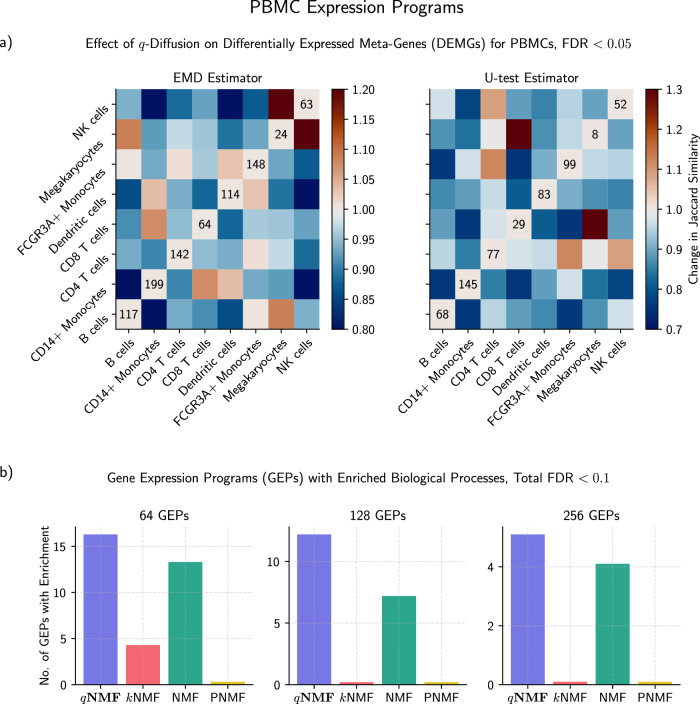
Exploring the PBMC expression programs, derived by *q*NMF and competing algorithms. **a** DEMGs between control and stimulated conditions are more unique to individual PBMC subtypes after *q*-diffused regularization is imposed on NMF. This effect is evidenced by the greater number of decreases (blue) in Jaccard similarity than increases (red) between the cell-type pairs of DEMGs. Decreases account for 75% and 89% of the off-diagonal entries, respectively. In the diagonals, we list the actual numbers of *q*-diffused DEMGs. **b** Total number of GEPs with at least one statistically enriched biological pathway. Competing NMF methods are shown for different amounts of GEPs, in powers of two. We analyzed *q*NMF, regularized NMF with a Seurat-style neighborhood graph (*k*NMF), typical NMF, and the recent Projective NMF (PNMF).

We also contrasted the gene expression programs (GEPs) identified from *q*-diffused nonnegative matrix factorization (*q*NMF) versus NMF. There were numerous differentially expressed meta-genes (DEMGs, see Method) between stimulated and control cells for each subtype, under both methods. DEMGs are like differentially expressed genes (DEGs), but for GEPs that carry statistically different weight between the two conditions. In Fig. 6a, we observe how DEMG commonality in cell-type pairs mostly decreased after *q*-diffused regularization. Overlap in DEMGs was measured through Jaccard similarity, which is normalized to the sizes of both sets. Figure 6a shows that the *q*-diffused DEMGs are more specific to particular cell types.

It is common to study the statistical enrichment of gene ontologies^35^ in GEPs as a way to validate their biological coherence^36^. Figure 6b tallies the number of enriched GEPs under competing methods and various settings. *q*NMF consistently outperforms the other three. The false discovery rate (FDR) was held below the critical threshold of 0.1. The threshold was higher than the more traditional 0.05 as the FDR spanned the combination of all ontologies and all GEPs.

### Third case study on brain structure

Spatial transcriptomics are gaining immense traction in biological and translational research^37,38^. For the purpose of this case study, we found ten samples of human cortical tissue extracted by MERFISH^39^. The immense spatial fidelity of this particular modality comes with the compromise in gene library size, limiting it to 4000 genes in the human samples. MERFISH is enabled by robust error-correcting barcodes to multiplex these gene readings^40^. Other spatial scRNAseq modalities like Visium^41^ have lower spatial resolutions for the trade-off of more genes.

A rather challenging aspect of analyzing brain tissue is the spatial nonlocality at the cellular level. In concrete terms, adjacent cells in the tissue may serve vastly different roles, like those of neurons, immune cells, and astrocytes. Therefore, classifying individual cells provides little information on larger-scale structure in the tissue. It is well known that the cortex has distinct functional layers. In the study that introduced this dataset^39^, cells could not be segmented by the known layers L1–L6 vis-à-vis their spatial transcriptomes. The original t-SNE visualization and clustering identified cell types, which were labeled by hand, but these only partly associated with specific layers.

In our experiments, we computed pairwise distance matrices between the small tissue regions termed rexels. These distances either came from the proposed local distributional segmentation (LDS) method (either *q*-diffused or Gaussian), or by *k*-Nearest Neighbors (*k*NN) on principal components of the rexels’ average expressions. Briefly, LDS takes into account the entire heterogeneous sample of cells in a rexel, in order to compare rexels as distributions of transcriptomes. In comparison to the other two case studies, the smaller samples in each rexel-rexel pairwise comparison justified a *k* = 16 neighborhood size for LDS. Finally the segmentation was performed by two popular algorithms^42^: Leiden community detection^26^ or hierarchical clustering with Ward’s linkage^43^.

#### Evaluations

**Fig. 7:**
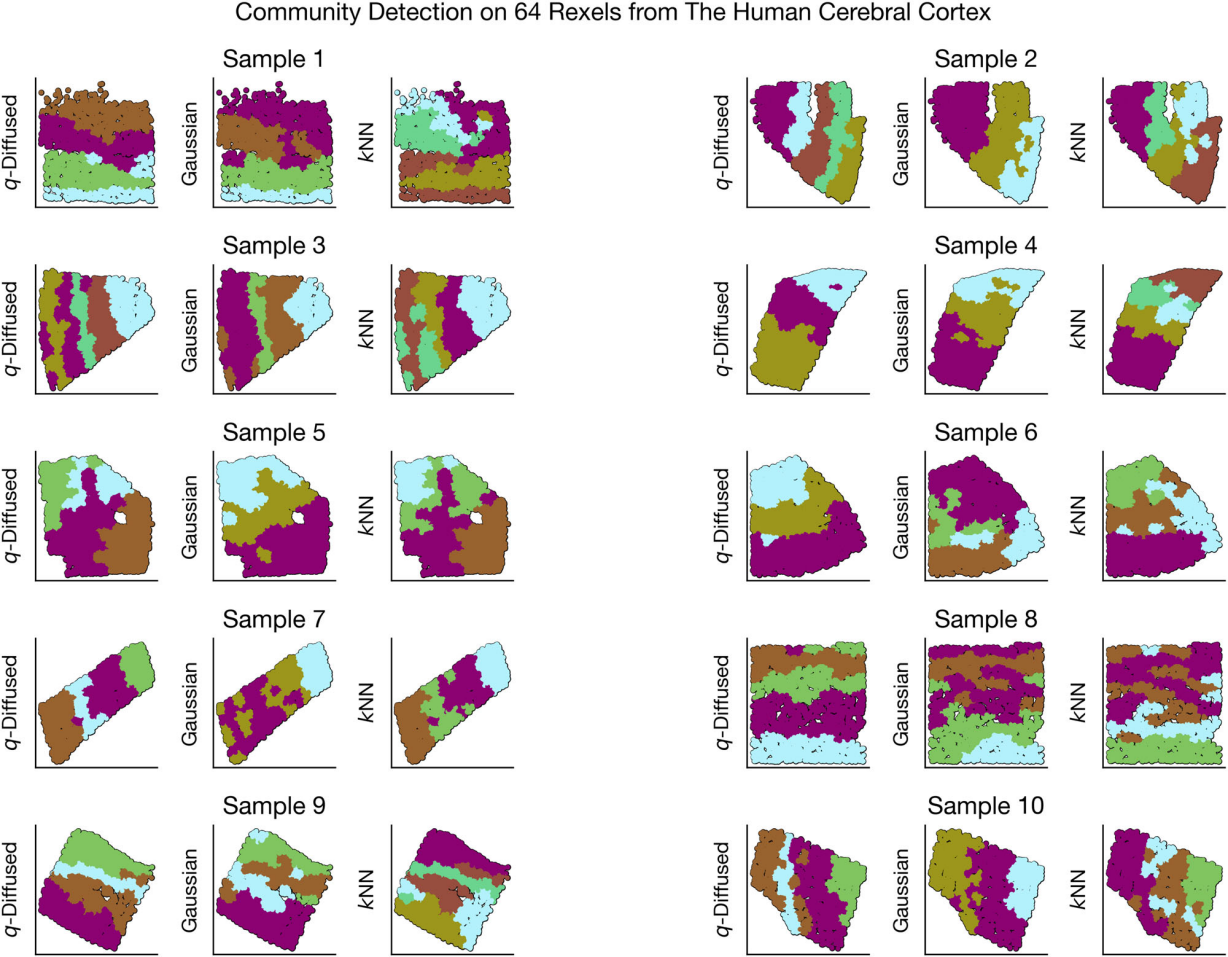
Side-by-side comparisons of local distributional segmentation with two alternatives, for ten human cortical tissue samples. Prior knowledge of the cerebral cortex compels the identification of parallal stripes to represent functional layers. Colors represent unsupervised clusters produced by the different methods, unaligned between competing versions.

**Fig. 8:**
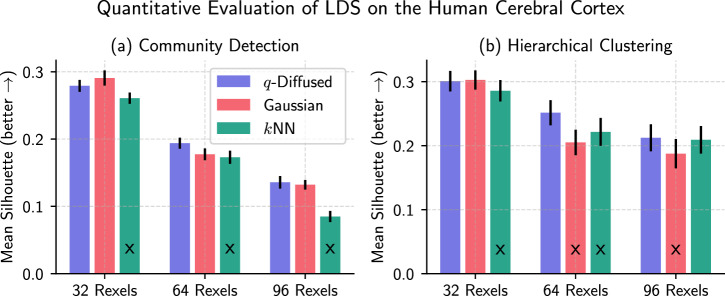
Quantifying the spatial contiguity of the rexel clusterings. Silhouette scores^44^ are compared across three methods for different numbers of rexels, using either (**a**) community detection, or (**b**) hierarchical clustering. Bars are equipped with standard errors. Those marked with `x' indicate a statistically significant improvement with *q*-diffusion over the Gaussian or *k*NN alternative as indicated by a *t*-test with *p* < 0.05. The opposite never occurs.

A comprehensive visual comparison of rexel clusterings is shown in Fig. 7. Clustering parameters were optimized by a grid search over the silhouette scores^44^ for each case. The objective in such a visual evaluation is to seek alignment with prior knowledge. The human cerebral cortex is organized into parallel functional layers^39,45^. It is apparent that the clusterings, which operate entirely on transcriptomic distributions and not pixel-wise spatial arrangements, ultimately tend to spatial contiguity. In more than half of the samples, notably Samples 1, 2, 3, 7, 8, and 9, parallel stripes appear to emerge clearly under *q*-diffused LDS. For a more quantitative assessment, we also contrasted the mean silhouette scores for various resolutions of rexel-lation (in multiples of 32 rexels.) The bars with significance markings in Fig. 8 suggest that *q*-diffused LDS was never significantly worse than the alternatives, and in many cases performed significantly better.

## Discussion

*q*-Diffusion led to demonstrable improvements in the multivariate analyses of differential effects between treatments in a clinical trial for mCRC (first case study). We identified groups of genes that could inform future treatment assignment through their prognostic implications. Additionally, it helped with classification and identificatio of GEPs for PBMCs with control and stimulated conditions (second case study). It also proved to be a necessary ingredient of LDS for recovering biologically relevant structures in human cortical tissue (third case study).

The granular nature of scRNAseq introduces sparsity and increases vulnerability to technical or biological noise^46^. Novel statistical methods like *q*-diffusion are required to intentionally handle the curse of dimensionality and its myriad of related effects^4^. Our results focused on findings that directly improved upon the state of the art for enhancing the utility of scRNAseq samples for foundational or clinical research.

The breadth of applications proposed for *q*-diffusion naturally intermingles with innumerable other approaches. For instance, the field is seeing renewed interest in framing scRNAseq problems in terms of optimal transport (OT)^47,48^. Multiple competing formulations exist for OT even in the generic problem of clustering cells^49,50^. The main point of disagreement is how to define distances between genes, which shape the OT distances between cells. Gene-to-gene distances could be defined through a corpus of ontologies, external reference datasets, or through their coexpressions in the same cells that are to be analyzed. A more fundamental limitation of OT is that cell-to-cell comparisons will always take quadratic time in the number of genes to evaluate, notwithstanding the Sinkhorn relaxation^51^. *q*-Diffusion takes linear time in the number of genes. The two orthogonal methodologies are both posed as improved geometries for transcriptomics. We compared them once in the second case study of this paper, but anticipate complementary use cases in the future.

The vast field of deep learning offers techniques^34,48,52,53^ that are complementary to, and perhaps synergistic with *q*-diffusion. We hope that *q*-diffusion could be harnessed upstream of a generative model’s objective function to inform its target geometry^54^.

Alternative cell-similarity metrics like the Spearman correlation appear more effective than a Euclidean distance in clustering^55^. Our benchmarks (second case study) were conducted against the standard analysis tools because to ad hoc insert correlation distances into an established pipeline would probably require adjusting the other hyperparameters. SC3 employs correlations, and was included in the benchmark. All of the benchmarked standard tools, which are close to the state of the art, are listed in Supplementary Table 1 alongside their key differences from *q*-diffusion.

Concretely, the benchmark on determining IFN-*γ* stimulation in PBMCs was remarkable because it represented a task that was more difficult than annotations of coarse cell types. Translational research often considers such workflows, in which certain broad phenotypes are sampled from patients and controls. The disease condition within a phenotype can be rather subtle. This is clearly the case for T cells pre-seroconversion of celiac disease in genetically predisposed individuals^56^. A similar challenge exists in beta or gamma cells from the pancreas of type-2 diabetic patients^57,58^.

The Tabula Sapiens benchmark evaluated *q*-diffusion against the dimensionality reductions supplied by the authors of the atlas, which were carefully selected for their data. Out of the four small-sample organ tissues tested, the *q*-diffused clusters vastly outperformed the baselines in alignment with ground-truth labels for three organs. The fourth organ, the liver, had 62% of its non-PBMC cells labeled as hepatocytes. The *q*-diffused clusters completely missed this separation, perhaps gravitating to other phenotypic differences in the tissue. Even though *q*-diffusion offers a novel and valuable perspective on scRNAseq datasets, it should be used in conjunction with more traditional analyses for a more complete picture of the phenotypes.

As for LDS with spatial scRNAseq, we address other approaches to spatial segmentation. A recent hidden Markov random field model for seqFISH^59^ needs a matching scRNAseq reference to disentangle cell-type variation from spatial variation, whereas the proposed LDS with MERFISH does not. DestVI^60^, a method based on variational autoencoders, requires external cell-type annotations. More comparable is an unsupervised method termed SSAM^61^, which detects cell-type signatures and then identifies spatial domains by comparing cell-type counts in sliding windows. We cluster on the basis of small tissue regions without discretizing to cell types. Also, we compare distributions of cellular transcriptomes rather than cell-type counts, by means of MMD. One of the baselines in the third case study was based on regional aggregates of principal components, reminiscent of SSAM sans the discretization.

The capability to reliably segment macro-scale structures from MERFISH should be contextualized in the broader field of brain research. Not only are the cells immensely heterogeneous even in small neighborhoods^39^, but signaling networks are highly reliant on spatial organization^62,63^. As spatiotemporal dimensions in scRNAseq samples become more accessible, there is immense promise in studying the process of memory formation^64^, among other phenomena in the brain. The first case study on *q*-diffusion was aimed at demonstrating outcomes-based biological and medical relevance by identifying potential mCRC biomarkers in a phase III clinical trial. It was important to isolate the findings to differential effects of treatments, or between treatments, in order to ensure that the tumor biomarkers possibly interacted specifically with the drug mechanism. Such findings could offer precision guidance for mCRC treatment and prognostication. Tumor angiogenesis—the growth of new blood vessels to supply oxygen and nutrients to cancer cells—is a critical process in the development and progression of CRC. Bevacizumab (bev), an anti-angiogenetic drug, is considered a standard treatment in combination with chemotherapy in first- or second-line. However, no predictive marker for bev efficacy is currently available for patient selection in the clinical setting. Through our analytical approach we were able to identify several genes associated with differential effects on bev treatment outcomes in mCRC. The majority of these genes are known to play a role in cancer, including CRC, and several are involved in angiogenesis-related pathways. However, we are the first to report a connection with bev efficacy in patients (except EREG, which had been previously identified^65^). Supplementary Discussion discusses the identified genes in detail.

This study has a few important limitations. First, *q*-diffusion arguably makes parametric assumptions on the data that might be restrictive in some use cases. Deep learning methods, on the other hand, offer more general avenues, but they notoriously demand large datasets and require extensive hyperparameter optimization. We also believe that *q*-diffusion is more interpretable than most solutions involving neural networks because despite its nonlinearities, *q*-diffused geometry is straightforward and supplements linear analyses like NMF.

Second, given the scope of this study, we did not consider batch-effect correction^66^ or alignment of disparate datasets. Presumably, various pre-existing tools can be coopted upstream of the analysis for this purpose. It is also of note that removal of batch effects also sacrifices some truly biological variation^52^, so it is important to conceive methodologies that can function without the corrections. Another limitation of *q*-diffusion is that nonlinearities are less directly interpretable, generally. We ameliorated this concern in the particular case of GEPs by formulating a *q*-diffused NMF to produce linear programs while guided by nonlinear dynamics. Finally, we note that there is room for improving the runtime efficiency of the algorithm evaluating the *q*-diffused kernel across all pairs of cells. With the Tabula Sapiens experiments serving as an example, which had an expansive library size of 58,870 genes, one server with four NVIDIA GeForce RTX 2080 Ti graphics cards took between 20 minutes (for the liver) to 3 hours (for the trachea) for the complete affinity matrix. A simple heuristic based on approximate nearest neighbors is likely to drastically improve runtime with minimal cost in accuracy.

Moving forward, we seek to study precisely when *q*-diffusion would be beneficial to a particular scRNAseq problem. Such an exploration would invite a careful selection algorithm for non-Euclidean deformation *q* and inner bandwidth *φ*, the two vital parameters to *q*-diffusion. At present, our custom software QDiffusion.jl has been released to the public with sufficient documentation for others to use.

## Methods

The core of our approach is a coexpression geometry for the transcriptome that overcomes the curse of dimensionality. Its name *q*-*diffused* points to the heavy inspiration from Tsallis statistics, which build on smoothly deformed *q*-*analogs* to many classical functions^67^. The *q*-diffused geometry can be framed as a deformation of Euclidean geometry, stemming from a *q*-deformed Gaussian function. In effect, a *q*-diffused norm differs from the Euclidean norm by introducing several interaction terms of increasing order, up to the entire dimensionality of the vector space. The consequence of these additional terms is that interactions spanning many variables are weighed heavily. Borrowing on historical notation, we parametrize the deformations by a single parameter *q*, in the range 1 < *q* ≤ 2 for our context, where the limit *q* → 1 reconstructs the original function for each *q*-analog. With $\alpha =:q-1\in \left(0,1\right]$, the *q*-diffused version of a Euclidean distance for vector *v* becomes

1 $${{}_{q} {||} {v} {||} } = \underbrace{v_1^2 + v_2^2 + v_3^2 + \ldots}_{{{{{{\mathrm{first}}}}}}\,{{{{{\mathrm{order}}}}}}\,({{{{{\mathrm{Euclidean}}}}}})} + \underbrace{\alpha(v_1^2 v_2^2 + v_1^2 v_3^2 + v_2^2 v_3^2 + \ldots)}_{{{{{{{\mathrm{second}}}}}}\,{{{{{\mathrm{order}}}}}}\,({{{{{\mathrm{pairs}}}}}})}}\ \\ + \ \underbrace{\alpha^2(v_1^2 v_2^2 v_3^2 + \ldots)}_{{{{{{{\mathrm{third}}}}}}\,{{{{{\mathrm{order}}}}}}}} + \underbrace{\alpha^3(\cdots) + \alpha^4(\cdots) + \ldots}_{{{{{{{\mathrm{higher}}}}}}\,{{{{{\mathrm{order}}}}}}}}$$

From this perspective, *α* can be viewed as a discount factor on interactions of increasing order. Figure 1 illustrates these interaction terms. Realizations of the method are described below, and details are in Supplementary Methods.

A plethora of nonlinear analytical methods rely on a *kernel*: a function that quantifies the proximity between two points in an observational space. The most common such kernel is the Gaussian kernel, sometimes called the *radial basis function*. The major benefit of drawing inspiration from the Tsallis framework is that it serves as a heuristic for constructing our *q*-diffused kernel. Fundamentally, all *q*-analogs are based on the *q*-exponential function, a polynomial that approximates the exponential and grows or decays slower for *q* > 1:

2 $${\exp }_{q}(x) : = {[1+(1-q)x]}_{+}^{\frac{1}{1-q}},\quad x\in {\mathbb{R}}, \\ \therefore \; {\log }_{q}(y) : = \frac{{y}^{1-q}-1}{1-q},\quad y\in (0,\infty ).$$

A kernel that decays by power law rather than exponentially makes up for the overall increase in distances after incorporating the interaction terms in Eq. (1). Generally the Gaussian kernel takes on the form of $f(x)=\exp -\beta {x}^{2}.$ In a multivariate setting, *x* is a vector norm. We shall outline the *q*-exponential’s link with the interaction terms in *x* discussed above. First, observe the following identity for isotropic Gaussian functions in two dimensions, *x* and *y*: $\exp (-\beta ({x}^{2}+{y}^{2}))=\exp (-\beta {x}^{2})\cdot \exp (-\beta {y}^{2})$. This does not hold in the *q*-analog. Specifically, we have instead

$${\exp }_{q}(-\beta {x}^{2})\cdot {\exp }_{q}(-\beta {y}^{2})={\exp }_{q}\left((-\beta {x}^{2}){\oplus }_{q}(-\beta {y}^{2})\right),\qquad \\ {{{{{{{\rm{where}}}}}}}}\quad a{\oplus }_{q}b : = a+b+(1-q)ab,$$

from which the interaction term, (1 − *q*)*ab* = − *β*^2^(*q* − 1)*x*^2^*y*^2^, emerges within the *q*-exponential. The binary ⊕ _*q*_ operator is termed a *q*-sum. Applying the *q*-sum recursively generates all orders of interaction. The following perfectly recreates the *q*-diffused norm ${}_{q} \left\| \cdot \right\|$ of Eq. (1), with *α* ↦ (*q* − 1): 

3 $${\prod }_{i = 1}^{m}{\exp }_{q}(-{v}_{i}^{2})= \,{\exp }_{q}\left(-{v}_{1}^{2}{\oplus }_{q}\left(-{v}_{2}^{2}{\oplus }_{q}(-{v}_{3}^{2}{\oplus }_{q}\cdots -{v}_{m}^{2})\right)\right) \\ = {\exp }_{q}({-}_{q}{\left\Vert v\right\Vert }^{2}).$$

**Fig. 9:**
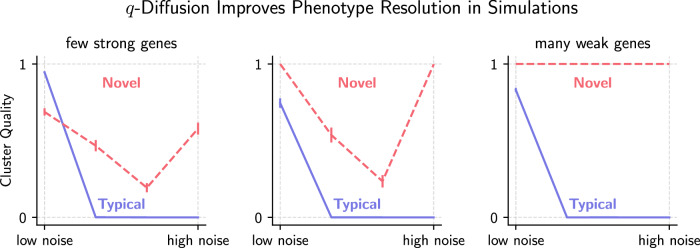
Simulated scRNAseq experiments for low-to-high technical/background noise and low-to-high diffusion across multiple genes of the phenotype separation. We showcase the superior ability of our proposed deformed geometry to recover two different phenotypes, compared to a typical undeformed geometry. Performance under various settings is assessed via the adjusted Rand index (ARI)^30^. Details and more illustrative figures are in Supplementary Methods.

The kernel above can be expressed as a product of univariate *q*-exponentials, or a single *q*-exponential with the *q*-sum quadratic form. The full *q*-diffused kernel incorporates two scaling terms, the inner and outer bandwidths, to control its behavior. The outer bandwidth breaks this duality of the multivariate kernel with the univariate-kernel product. Figure 9 as well as Supplementary Figs. 1 and 2 highlight the desirable properties of this construction.

### Definition 1

The *q*-diffused kernel on vector $v\in {{\mathbb{R}}}^{m}$, with outer bandwidth *ρ* > 0 and inner bandwidth *φ* > 0, is given by

$$f(v) : = \, {\exp }_{q} \left(-\frac{{\,\!}_q{\left\| {\varphi }^{-1}v \right\| }^{2}}{{\rho }^{2}}\right).$$

We always employ the *q*-diffused kernel at an adaptive resolution, where the outer bandwidth *ρ*(*k*) is set to the *k*th nearest *q*-diffused norm in a cell’s neighborhood. This *k*NN parameter is set from the scope of the problem; for instance, when performing community detection, this *k* takes on the same role as in the *k*NN graph construction that is common in other methods. Notably, our adaptive approach follows that of PHATE^13^. The kernel is truly anisotropic, in that *ρ*(*k*) depends on the origin point. So *f*(*a*, *b*) := *f*(*b* − *a*) with *ρ*(*k*) scaled by the neighborhood of *a*.

### The inner bandwidth

The inner bandwidth *φ* acts as a soft threshold for the magnitude of interactions. Gene differences exceeding *φ* in magnitude tend to cascade more strongly up the higher-order terms of the *q*-diffused norm (Eq. (1)). It is widely recognized that the variance (post-normalization) of expressions across a gene library is massively imbalanced: an instance of Pareto’s principle at work in nature. This is the reason that common analysis pipelines perform aggressive feature selection based on variance or dispersion, and sometimes rescale the remaining genes to equalize their importance. One of the main motivations behind our work is the belief that those preprocessing steps are too crude for complex gene processes. First, most genes are discarded; second, the rescaling of those remaining inevitably distorts their processes. It is often seen as necessary in order to analyze low-expression but important genes like transcription factors^68^. Our approach avoids the aforementioned pitfalls via this inner-bandwidth mechanism, a knob that allows us to navigate the spectrum of multiscale processes. Roughly, expression magnitudes far above this knob are amplified and those below are discounted.

We devised a simple heuristic for choosing the inner bandwidth, and adhered to it for all the results presented. For each gene, we computed the average pairwise squared distance across cells. Then we picked a quantile of these gene scatters for the inner bandwidth. Since the variance is concentrated to just a few genes, ideally one could choose a relatively high quantile like 90% and still pay attention to most genes. However, for simplicity, we opted with the 50% (median) quantile. This endows us with a setting for the inner bandwidth prior to analysis.

#### Choosing the deformation parameter

As there was both a wide (in terms of clustering, factorization, embedding) and deep (for competing methods) set of comparisons to perform against the novel *q*-diffused framework, we chose to narrow the space by considering only *q* = 1.2 for *q*-diffusion. See Supplementary Methods for an exploration on the effect of other settings.

### Using the kernel

After filtering and basic normalization, scRNAseq data consist of *n* observed cells with *m* sparse gene measurements each. We denote them as a matrix with cell column vectors $X=[{x}^{(1)}\,{x}^{(2)}\,\ldots \,{x}^{(n)}]\in {{\mathbb{R}}}^{m\times n}$. The most common methods of analysis involve *clustering* or *embedding*, where cells are categorized into apparent phenotypes, and plotted in a low-dimensional space representing their semantics or relations. Figure 1 shows how *q*-diffusion improves on common analyses.

All the augmentations to existing methodologies that we explored involved some form of an affinity matrix $A\in {{\mathbb{R}}}^{n\times n}$, filled with kernel evaluations between pairs of data points in *X*. As with PHATE^13^, we symmetrified the anisotropic matrix arithmetically:

4 $${\tilde{A}}_{i,j} : = f({x}^{(i)},{x}^{(j)}),\qquad A : = (\tilde{A}+{\tilde{A}}^{T})/2.$$

The recursive structure of Eq. (1) revealed a divide-and-conquer algorithm, which was implemented and released as open-source software to compute these matrices while taking advantage of massively parallel GPUs using the CUDA platform. Numerical stability is maintained by performing the computations in a logarithmically transformed space.

### Community detection

Considering the affinity matrix as a weighted, undirected graph adjacency matrix (by subtracting the diagonal,) we performed community detection on the basis of the state-of-the-art Leiden algorithm optimizing for modularity^26,42^. This was benchmarked in the second case study of this paper.

### Gene expression programs (GEPs)

We also adopted nonnegative matrix factorization (NMF), of demonstrated efficacy in scRNAseq studies^23^. The estimated basis vectors of such a method are often called gene expression programs (GEPs) or meta-genes because they capture sparse sets of genes that express together and are likely coregulated. Each cell is deconstructed into a set of combination weights on the GEPs. This factorization often serves as the first step to data-driven cell phenotyping^15,69^. For the sake of biological coherence, it is valuable to require a cell’s GEP weights to be similar to those of nearby cells in the transcriptomic space. We consider, in particular, the affinity matrix that is induced by our *q*-diffused kernel. The structure encoded in this matrix is highly nonlinear, and even though the GEPs themselves are linear, we may attempt to guide them by the nonlinear structure^70^ in order to improve downstream results.

The resultant programs between *q*NMF and NMF GEPs were qualitatively similar, partly because they were always initialized with the same random seed, but mostly because NMF picked up the strong coexpression patterns. The most heavily weighted genes in each GEP were almost equivalent between their two versions. What differed were the precise weightings, which trickled into subsequent quantitative findings.

### Local distributional segmentation (LDS)

We developed an approach to spatial segmentation of a tissue informed by spatially resolved transcriptomics for the third case study in this paper. Undoubtedly, an unsupervised technique could massively aid in the discovery of structures^71,72^ in tissue samples. Functional segmentation of a tissue must be performed at a scale coarser than that of the single cell, yet still informed by cellular heterogeneity. Still, the scale must be granular enough to preserve the intricacies revealed by MERFISH. We chose to operate on small neighborhood groupings of cells, termed *rexels* for region-level pixels. Concretely, each tissue sample was divided into an approximate Voronoi parcellization by repeated *k*-means.

We hypothesized that categorizing rexels through their *distributions* of heterogeneous cells would provide stronger functional information than any neighborhood-level aggregation. A rexel is expected to be a sample of neurons, immune, and auxilliary cells, which together make up the functionality of that part of the brain. Our novel LDS approach entailed the computation of distances between all rexel pairs through the maximum mean discrepancy (MMD)^73^, a recently popular kernel-based measure for comparing two multivariate samples^74^. Intuitively, to compare two sets of high-dimensional points, MMD averages the pairwise kernel values *within* each set and contrasts them with the averaged pairwise kernel values *between* the sets. We experimented with our *q*-diffused kernel and a more typical Gaussian baseline for MMD. Moreover, we compared with a simpler segmentation method on the basis of neighborhood aggregates, where rexels were represented by the gene expressions averaged over their constituent cells.

### Dimensionality reduction

Researchers are rather concerned over the amount of unseen distortion of global-structure biological patterns in the most popular embedding methods for scRNAseq^75^. For this reason, t-SNE and UMAP are often relegated to mere visualization. A lesser known, albeit well founded and characterized^14^ alternative is PHATE^13^. We augmented PHATE with the *q*-diffused kernel of Definition 1, symmetrified by Eq. (4). We harnessed the *q*-diffused PHATE embedding for a task beyond mere visualization: to screen important genes in mCRC, and estimate a biomarker for clinical-trial patients.

### Differential effects of latent variables

Groups of latent variables were tested in the first case study of this paper, on mCRC for identifying transcriptomic interactions with treatments onto patient outcomes. The effects of the latent varibles were modeled as Cox proportional hazards^76^.

Two sets of latent variables were identified with the help of *q*-diffusion. For each set, their regression coefficients on outcomes were contrasted between the treatment cohorts. Each pair of coefficients for the same latent variable, corresponding to its effects under the two treatments, was tested for a nonzero difference using the asymptotic normal approximation^77^. The *z*-tests were performed on the basis of variance estimates through observed Fisher informations, which is established practice^78^, and then corrected for multiple testing^79^. Statistically significant differences, especially with opposing signs, signified differential effects from these latent variables. The dichotomy in an effect’s value would suggest a biomarker for a possibly causal interaction with a treatment, since treatment-cohort assignments were fully randomized.

For *q*NMF, we limited our investigation a priori to 16 potentially novel latent variables. This amount was chosen as a round power of two, and probably the maximal supported by the sample size of 557 in the clinical trial. We avoided further explorations of different amounts as they would risk a loss of statistical power^80^. As some GEP weights transferred to patients were entirely zero or entirely nonzero, we also screened for GEP weights with sparsity no less than 5% and no greater than 95% for our tests of significance.

The latent variables were inferred from an scRNAseq “atlas” reference dataset^15^. The stage-4 portion of the atlas was selected, matching the conditions of the patients in a clinical trial, so that insights from the atlas could be translated directly to the patients. This clinical trial recorded patient outcomes for two different treatments, alongside bulk RNA profiles from tumors. As presented in Fig. 2, the inferred latent variables consisted of two sets of 16 gene expression programs and branching gene components. Translation of atlas-inferred latent variables onto patients’ RNA profiles was performed by linear deconvolutions with the estimated single-cell programs or components.

### Differentially expressed genes (DEGs)

A multitude of techniques exist for identifying differentially expressed genes (DEGs). These approaches can transfer to the meta-genes^59^ revealed by whole GEPs, yielding differentially expressed meta-genes (DEMGs). A recent benchmark on single-cell DEG identification suggested that the earth-mover’s distance (EMD)^81^ offers the best tradeoff between precision and recall^82^. The Mann-Whitney U test (or Wilcoxon rank-sum test) is a simpler statistic often used for DEGs^83^. A study on bulk RNA sequencing provided some evidence that more complex techniques tend to exaggerate the false positives^84^. Hence, the EMD permutation tests and U-tests for differential expression facilitated assessments of GEP estimators for isolating DEMGs. The Benjamini–Hochberg^79^ procedure corrected for multiple testing by controlling the false discovery rate (FDR).

### Reporting summary

Further information on research design is available in the Nature Portfolio Reporting Summary linked to this article.

### Supplementary information

Supplementary Information

Description of Supplementary Materials

Reporting Summary

supplementary data

## Data Availability

All datasets considered in this study have been previously reported in the literature. Accession codes or links are provided for all datasets besides the clinical trial: the Human Colon Cancer Atlas can be accessed at GEO: GSE178341; the PBMC benchmark at GEO: GSE96583; the Tabula Sapiens benchmark at 10.6084/m9.figshare.14267219.v5; the human MERFISH sample at 10.5061/dryad.x3ffbg7mw. For the CALGB/SWOG 80405 clinical trial, a summary of clinical and genomic data will be made available upon reasonable request.

## Code availability

Please visit https://github.com/marmarelis/QDiffusion.jl for access to the Julia package.
