# Supplementary material for: q-Diffusion leverages the full dimensionality of gene coexpression in single-cell transcriptomics
Source: Commun Biol. 2024 Apr 2;7:400. doi: 10.1038/s42003-024-06104-w (PMC11255321; doi:10.1038/s42003-024-06104-w)
Supplement: Supplementary file 1 — Supplementary Information [file 42003_2024_6104_MOESM1_ESM.pdf]

Supplementary Methods

Details Behind The Synthetic Example (Figure 9)

We simulated a hundred distinct scenarios and aggregated their results for Figure 9. In each case, two different *pseudo*-phenotypes were drawn independently from a thousand-dimensional Dirichlet distribution with one uniform parameter, α_D. The background or noise expression level was controlled by another parameter, α_N, which balanced an arithmetic mixture between the Dirichlet-drawn vector and ones. In other words, one phenotype was internally constructed as

$$X = \max\left\{(1-\alpha_N)Y + \alpha_N \mathbf{1},\ 0.9\right\}, \qquad \text{where} \quad Y \sim \text{Dirichlet}\left([\alpha_D]^{1:1000}\right).$$

The random vector X represents the gene activities of a phenotype. Higher noise levels entail a greater overall expression of all the genes, indiscriminately. This conflicts with phenotype resolution. Observations were modeled as univariate negative binomial distributions with dispersion parameter $r = 10^3$. The negative binomial fits scRNAseq empirically well [1]. Before analysis, observations were log-normalized as with real data.

We benchmarked community detection on either the q-diffused kernel, or the Gaussian kernel with eight principal components. Communities were compared against the binary ground-truth phenotypes by the Rand index. In Figure 9, the three charts ranging from "few strong genes" to "many weak genes" had $\log_{10}\alpha_D = -4, -3, -2$, respectively. In each chart, the range from "low noise" to "high noise" consisted of the settings $\log_{10}\alpha_N = -5, -4, -3, -2$. We note that the curious increase in accuracy for q-diffusion at the highest noise level is most likely an artifact of the normalization method. We also consider it a testament to the promise that this method holds.

More Details on q-Diffusion

The q-diffused kernel is a deformation of the classic Gaussian radial basis function, placing a power-law decay on each gene term. Our approach draws inspiration from nonextensive entropies [2] and nonlinear Fokker-Planck equations [3].

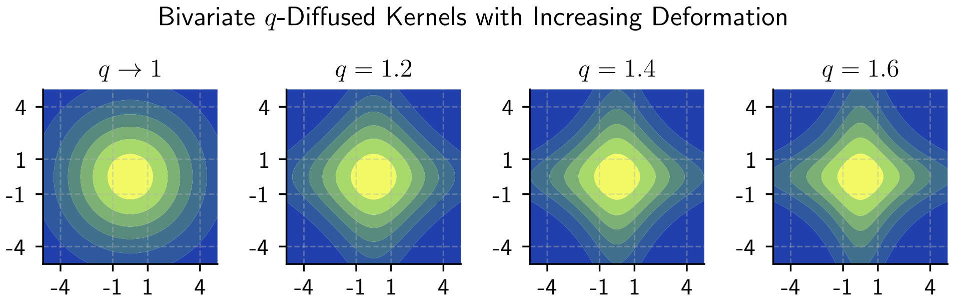

Supplementary Figure 1. Contours of bivariate q-diffused kernels, demonstrating their concavity, which incidentally violates the positive semidefinite property. The tick marks indicate the inner bandwidth that is set to 1, with the outer bandwidth set to 4 for the purpose of illustration. Throughout the paper, q-diffusion refers to $q = 1.2$ specifically.

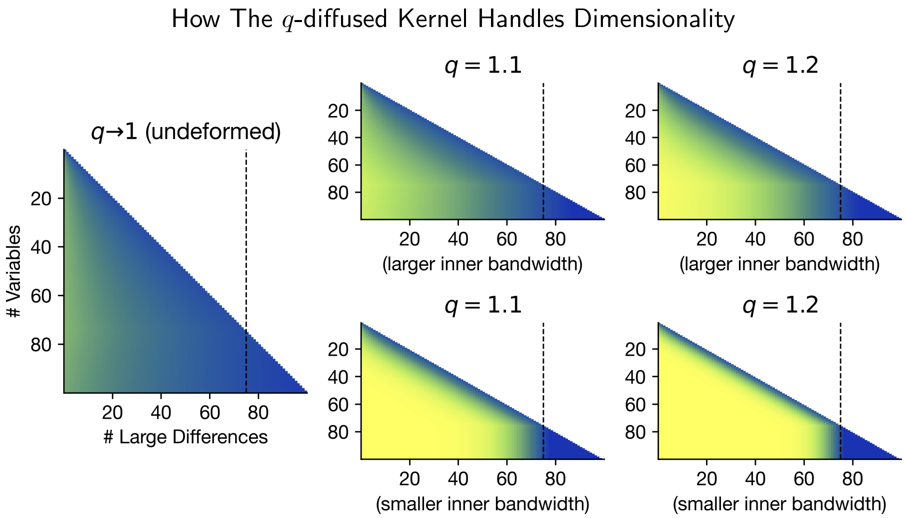

Supplementary Figure 2. Kernel values when different numbers of variables have large displacements, at various dimensionalities. Colors are scaled linearly in $[0.25, 1.00]$. Bandwidth is set to the distance equivalent of 75 variables with large differences (marked by vertical dashed line). The q-diffused kernel's behavior depends on relative scaling with the inner bandwidth, as showcased with the small and large versions plotted.

Implementation Details

Preprocessing. All scRNAseq datasets considered in this study were log-normalized with an assumed transcript throughput of 10^4 per cell, as is typical and often considered standard [4].

Regularization hyperparameter for qNMF. The q-diffused NMF introduced one additional hyperparameter. Namely, one must select how to weigh the reconstruction error against the q-diffused regularization. It is unreasonable to optimize this weighting on the basis of insample reconstruction because the regularizer always worsens this. A feasible route for insample model selection could be through information criteria like the AIC or BIC [5] on the *effective* sizes or degrees of freedom in the GEPs. For our purpose in the mCRC case study, model fitness depends on downstream utility. Validating GEPs on the smaller sample of clinical-trial patients would ruin the statistical significance. Therefore, we opted for a scale-invariant weighting heuristic that eliminated the need for hyperparameter selection. Our heuristic, available in the source code, weighs one unit of reconstruction error equally against one unit of discrepancy in meta-gene expression between immediate neighbors in the q-diffused geometry.

Finding active genes in a GEP. Active genes in a GEP were considered those with effectively nonzero ($> 10^{-3}$) weight. NMF and qNMF produce rather sparse GEPs [6], and downstream processing is relatively insensitive to the precise threshold.

Gene ontology enrichment. The PANTHER system [7] was employed to investigate the overrepresentation [8] of gene ontologies from specific biological processes in each GEP.

Deconvolution of GEPs in bulk RNA. Similarly to the approach taken with CIBERSORTx [9], we perform nonnegative least squares for deconvolution of GEP estimates onto bulk RNA samples.

Theoretical Motivation

The terminology of q-diffusion relates to the geometry's effect, in practice, to identify processes that diffuse across many variables. Separately, its theoretical underpinnings relate to heat diffusion [10].

Briefly, we contextualize this work in the realm of Riemmanian manifolds [11], [12]. A Riemmanian metric operates on locally linear tangent spaces on a manifold. It manifests as a quadratic form, hence the local behavior of any Riemmanian manifold can exhibit curvatures described by bilinear terms at most. Intriguingly, the q-diffused geometry breaks out of that framework and incorporates terms of much greater order.

Physical Motivation

The Fokker-Planck equation describes the evolution of an ensemble of infinitesimal particles. Individually, a particle is governed by a stochastic Langevin equation. The Fokker-Planck equation leads to a probability density function of these particles, spreading wider with time. Reasoning about a particular *microscopic* behavior due to some Langevin equation sheds light onto the mechanics of its corresponding *macroscopic* law [13]. A general Itô-Langevin equation with scalar x is written as

$$\frac{\mathrm{d}x}{\mathrm{d}t} = \mu(x,t) + g(x,t)\eta(t) \tag{1}$$

with stochastic elements contained in the noise $\eta(t)$, with $\mathbb{E}\,\eta(t) = 0$. A particle following such a kinematic obeys, on average, a probability density $f(x,t)$ over a spatial dimension x, changing with time t, possibly conforming to a corresponding Fokker-Planck equation. Should $\eta(t)$ be a Wiener process (Gaussian white noise,) the linear case is recovered. The heat equation that arises in kernel methods is closely tied to the driftless (with $\mu = 0$) version thereof. This case allows for the solution to an arbitrary initial-value problem to take the form of a convolution by the so-called *heat kernel*, which looks like a Gaussian function with scale $\propto \sqrt{t}$.

A remarkable nonlinear framing of the Fokker-Planck equation is given by [3]

$$\partial_t f = -\partial_x(\mu f) + Q\partial_x^2(f^\nu) \tag{2}$$

with constants Q and $\nu \neq 1$. The only way for this form to be compatible with Equation 1 is for the microscopic diffusion function g to be coupled with the macroscopic by $g(x,t) = Qf^{\nu-1}(x,t)$. This fact implies that there is an interaction among the particles, such that motion of the ensemble influences individual jump sizes. That phenomenon is reflected in the general solution (with linear drift,) which is a time-dependent q-Gaussian with $\nu = 2 - q$:

$$f(x,t) = Z(t)^{-1}[1 - \beta(t)(1-q)(x - x_M(t))^2]_+^{\frac{1}{1-q}}$$

and certain functions $Z(t), x_M(t), \beta(t)$. When $\nu < 1, q > 1$, and $g(x,t)$ depends on $f(x,t)$ taken to a negative power. In other words, jump sizes increase as a particle reaches the extremities of the distribution. The $q < 1$ case has been studied extensively in the context of the porous-medium equation [e.g. 14], which yields narrow tails. These univariate equations (in x) are a possible framing for how q-diffused dynamics could arise in nature.

Alternative Motivations

Proposition 1. *The q-diffused kernel around $q \to 1^+$ behaves as*

$$\prod_{i=1}^{m} \exp_q\left(-x_i^2\right) = \exp\left(-\|x\|^2\right) \cdot \left[1 + (q-1)\frac{1}{2}\sum_{i=1}^{m} x_i^4\right] + O(q-1)^2.$$

Proposition 1 suggests that there exists a sort of compensatory mechanism in the q-diffused kernel, propping up the kernel's value when it would otherwise be low due to a few high entries (hence with strong fourth powers.) It may also be helpful to think of the q-diffused kernel in terms of its associated distance-like function, termed q-Euclidean.

Definition 1. The *q-Euclidean pseudonorm* of a vector $x \in \mathbb{R}^m$ is ${}_q\|x\| \coloneqq \left[-\bigoplus_{i=1}^{m}(-x_i^2)\right]^{1/2}$. It is properly considered a pseudonorm because it does not obey the triangle inequality.

Lemma 1 (local asymptotic)**.** *The squared q-Euclidean pseudonorm, within a ball of radius $O(\sqrt{\varepsilon})$ in $\mathbb{R}^m$ for sufficiently small ε, may be approximated by the squared Euclidean norm up to the order ε^2. In fact, locally,* $\bigoplus_{i=1}^{m} \pm x_i^2 = \pm\|x\|_2^2 + O(\varepsilon^2)$.

Lemma 2 (global bound)**.** *Anywhere in $\mathbb{R}^m$, the q-Euclidean pseudonorm is bounded below by the Euclidean norm:* ${}_q\|x\|^2 \geq \|x\|^2$, *for $q > 1$.*

Note that the q-diffused kernel is not positive (semi)definite, posing challenges with incorporating Reproducing Kernel Hilbert Space (RKHS) theory. We plan to investigate the theoretical implications in future work.

Supplementary Results

Solution	Preprocessing	Dimensionality	Orders of Interaction	Cell Distances	Neighborhoods
<u>q-Diffusion</u>	normalization	all genes	all $\gg 2$	power law non-Euclidean	q-diffused
Seurat	normalization, **feature selection**, standardization	a few principal components	2	Euclidean	Shared Nearest Neighbors (SNN)
Scanpy	normalization, **feature selection**, standardization	a few principal components	2	Euclidean	UMAP or DM
SC3	normalization, **feature selection**	a few principal components	2	Euclidean/ Pearson/ Spearman	k-means
Monocle	normalization	a few principal components	2	Euclidean	embedding SNN (e.g. in UMAP)
Optimal Transport	normalization	all genes	N/A	through gene-gene distances	SNN

Supplementary Table 1. Our method contrasted against the state of the art in cell clustering and dimensionality reduction. We list the default setting for packages that expose a variety of options. Blue: clear improvements; red: drawbacks.

Details for Case Study on Colon Cancer

Local Intrinsic Dimensionality (LID). The LID for Figure 3 was estimated by a maximum-likelihood method [15], computed over a point's $2^9 = 512$ nearest neighbors. The heatmaps were smoothed for the purpose of interpretation by taking a median "vote" over $2^{11} = 2,048$ neighbors for the value of each point. There were $17,362$ points in total.

Details for Case Study on Clustering

This case study presented a more nuanced task than the well-characterized PBMC classification problem. We believe it is representative of real studies into the more granular phenotypes that could inform the outcome of a disease. A specific example is how the effect of IFN-γ on PBMCs is a risk factor for COVID-19 [17], [18].

q-Diffusion was applied with $k = 64$ for the neighborhood sizes. Standard (or analogous, when there is no "standard") neighborhood settings were used on all the competing methods. The benchmark included the

most popular and current software packages for single-cell clustering [19], namely Seurat [4], Monocle [20], Scanpy [21], SC3 [22], as well as optimal transport [23]. For all but SC3, as explained below, we evaluated the variety of ways that neighborhood graphs were constructed across all PBMCs. Then, we subsetted the graphs by cell type and executed the Leiden community detection algorithm [24]. SC3 could only output clusters directly for each subtype. Optimal transport, which has yet to attain a standard operating procedure [e.g. 25] and is rather computationally expensive [26], was attempted with reasonable settings. Previously proposed methods like SNN-Cliq [27] and PhenoGraph [28] were not included because they inspired the latest versions of packages like Seurat.

The Optimal Transport (OT) Baseline. As mentioned in the second case study of the paper, there is no established toolkit for OT-assisted clustering as of the time of this writing, and we had to construct a simple baseline. At a high level we estimated gene-to-gene distances internally, through Euclidean distances between their log-normalized expressions across the cells [23]. Then we renormalized expressions to the probability simplex and ran the Sinkhorn algorithm with regularization $\varepsilon = 1$. For computational reasons, we selected $4,000$ features using the Seurat dispersion metric, where Seurat typically selects $2,000$ features. As we sought the $k = 64$ nearest neighbors due to OT for the purpose of constructing a graph and performing community detection, we restricted the search to the $2^9 = 512$ Seurat neighbors—in other words, Euclidean in the principal components—of each point. These Seurat neighbors were filtered down to the nearest 12.5% in terms of OT.

Details for Case Study on Brain Structure

Grid search for LDS clustering. Clustering parameters were optimized by silhouette-score sample means over a simple grid. The search for community detection was conducted over $k = 5, 6, 7, 8, 9, 10$ nearest neighbors for the directed graph construction. For hierarchical clustering, we tried $k = 2, 3, 4, 5$ partitions.

Supplementary Discussion

Among the individual genes associated with improved outcomes under bevacizumab, VAV3 plays an important role in tumor angiogenesis and is associated with CRC growth and metastatic spread through PI3K-AKT signaling [29]. SLC2A8 is part of the intracellular glucose transporters solute carrier 2A family, several members of which have been recently connected to angiogenesis, as higher glucose uptake and glycolysis have been linked to survival in bevacizumab-resistant tumors[30]–[32]. SPATA13 (also known as Asef2) interacts directly with APC and promotes colorectal tumorigenesis, progression and invasion [33]–[35]. NHLRC3 has been recently included in a prognostic model of seven-gene signature to predict CRC survival [36]. This gene encodes for a protein involved in the process of ubiquitination which modulates many cellular processes and signaling pathways. TRPC4AP and RNF114 are also involved in the ubiquitination of various substrates. TRPC4AP specifically mediates the degradation of MYC and participates in the activation of NFKB1 and JNK. MYC is an extremely important oncogene which plays a major role in cancer and contributes to tumor cell invasion, migration and angiogenesis [37]. On the other hand, NFKB activation in CRC also plays a central role in promoting angiogenesis and tumor growth [38]. RNF114, an E3 ubiquitin ligase, has been shown to negatively regulate NFKB signaling and to modulate T-cell activation and apoptosis, which could influence the balance of the immune tumor microenvironment (TME) [39]. COMMD7 also regulates NFKB signaling [40]. SETDB1, a histone methyltransferase, promotes CRC progression via epigenetically silencing of p21 expression [41]. Studies have reported significantly higher SETDB2 frameshift mutations in MSI-H CRC. CC2D1A has been recently reported as part of a model predicting sensitivity to anti-EGFR treatment in KRAS-wildtype CRC [42]. This gene is also involved in several pathways, including NFKB, Akt, PKA and Notch signaling. Notch signaling and its crosstalk with other signaling pathways is pivotal in cancer progression, including tumor angiogenesis and cancer stem cell expansion [43]. Another of our significant genes, CBFA2T2 (or MTGR1) is a Notch signaling inhibitor [44]. Conversely, ASPH which is associated to reduced benefit from bevacizumab in our analysis, can promote Notch signaling and has been related to tumor invasion and reduced patient survival in CRC [45]. PLCG1 has also been identified as a novel mechanism of anti-EGFR resistance in RAS wild-type CRC [46]. PIGU, MANBAL, and AAR2 have been found to be

associated with KRAS Mutation in CRC, where PIGU low expression conferred a poor prognosis [47], [48]. TTI1 encodes for a protein involved in DNA damage response and part of the mTOR complex, a signaling mediator which plays a key role in angiogenesis modulation [49], [50], and has been reported to correlate with CRC proliferation and metastasis [51], [52]. Oxidative stress is another crucial pathway involved in cancer dynamics, including mechanisms of drug resistance and angiogenesis modulation [53]. Among our significant genes GSS [54], [55], OSER1 [56], and PRDX5 [57] are all involved in cellular response to oxidative stress. Additionally, PRDX5 promotes epithelial-mesenchymal transition (EMT) in CRC, which in turn stimulates tumor angiogenesis [58], [59]. ACOT8 has a role in β-oxidation during the metabolism of fatty acids and has been reported as a part of prognostic signatures based on lipid metabolism in CRC [60], [61]. DPM1 has been found to be overexpressed in CRC [62] and has been related to cell proliferation and enhanced angiogenesis [63], [64]. EREG (epiregulin) is the only gene that have been previously reported as a predictive biomarker for bevacizumab treatment in mCRC [65]. Consistently with our results, higher EREG expression was associated with longer survival in patients receiving bevacizumab plus chemotherapy.

Among the genes whose high expression is associated with worse outcome under bevacizumab treatment we found two members of the S100 family of proteins, S100A14 and S100A13. This protein family is well characterized for its role in cancer. S100A14 is involved in CRC cell growth and motility, and its expression has been reported to be inversely correlated with CRC progression [66]. S100A13 has pro-angiogenic functions reported in melanoma and astrocytic gliomas [67]. HMGA1 is a target of the oncogene MYC and promotes CRC development through Wnt signaling, another central signaling pathway in CRC biology [68]. It also has a pro-angiogenic activity [69], [70]. HMGA1 has been found to be overexpressed in CRC and has also been connected to metabolic pathways which promote cancer progression [71]. AGR3 promotes CRC stemness via Wnt/β-catenin signalling [72], which has been linked to treatment resistance. On the other hand, increased CTNNBL1 (β-catenin like protein 1) was associated with better bevacizumab treatment outcomes. Finally, CD163 encodes for a receptor expressed in monocytes and macrophages. CD163 positive macrophages in the TME have a M2 polarization and promote inflammation, vascular permeability and angiogenesis [73]. The infiltration of CD163 positive macrophages is a negative prognostic biomarker in CRC patients [74], [75]. Importantly, M2 macrophage polarization has been connected to bevacizumab resistance.

Proofs

Proof of Proposition 1. First we shall prove that $\exp_q(-x_i^2) = \exp(-x_i^2)\left[1+\frac{q-1}{2}x_i^4\right]+O(q-1)^2$. Let $\alpha = 1-q$ and $u = -x_i^2$. Then $\exp_q(u) = (1+\alpha u)^{1/\alpha}$, for $\alpha < 0$. We proceed by constructing a Taylor expansion at the limit $\alpha \to 0$: by definition of the exponential, $\lim_{\alpha\to 0}\exp_q(u) = \exp(u)$. Then we take the first derivative.

$$\begin{aligned}
\frac{\partial}{\partial\alpha}\exp_q(u) &= \frac{\partial}{\partial\alpha}\exp\{\alpha^{-1}\log(1+\alpha u)\} = \left[\frac{1}{\alpha}\left(\frac{u}{1+\alpha u}\right) - \frac{1}{\alpha^2}\log(1+\alpha u)\right](1+\alpha u)^{1/\alpha}. \\
\lim_{\alpha\to 0}\frac{\partial}{\partial\alpha}\exp_q(u) &= \left[\lim_{\alpha\to 0}\frac{\frac{\alpha u}{1+\alpha u} - \log(1+\alpha u)}{\alpha^2}\right]\exp(u) = \left[\lim_{\alpha\to 0}\frac{\frac{u}{1+\alpha u} - \frac{\alpha u^2}{(1+\alpha u)^2} - \frac{u}{1+\alpha u}}{2\alpha}\right]\exp(u) \\
&= \left[\lim_{\alpha\to 0} -\frac{u^2}{2(1+\alpha u)^2}\right]\exp(u) = -\frac{1}{2}u^2\exp(u) = \frac{1}{2}x_i^4\exp(-x_i^2), \qquad \text{by L'Hôpital's rule.}
\end{aligned}$$

Hence the univariate case is proven (for $m = 1$.) The multivariate cases follow by induction: chaining multiple $\exp_q(-x_i^2)$ terms yields a Gaussian function with unit and x_i^4 factors, and everything else of order $(q-1)^2$ and above. □

Proof of Lemma 1. Borrowing notation from Equation 3,

$$\begin{aligned}
\bigoplus_{i=1}^{m}(\pm x_i^2) &= \sum_{l=1}^{m}\sum_{I_l}(1-q)^{l-1}(\pm x_{i_1}^2)(\pm x_{i_2}^2)\cdots(\pm x_{i_l}^2) \\
&= \sum_{l=1}^{m}(\pm 1)^l(1-q)^{l-1}\sum_{I_l}x_{i_1}^2 x_{i_2}^2\cdots x_{i_l}^2 \\
&\le \sum_{l=1}^{m}(\pm 1)^l(1-q)^{l-1}\sum_{I_l}\exp\left\{l\cdot\log\left(\frac{x_{i_1}^2+x_{i_2}^2+\ldots x_{i_l}^2}{l}\right)\right\}
\end{aligned}$$

due to Jensen's inequality on

$$\log\left(x_{i_1}^2 x_{i_2}^2\cdots x_{i_l}^2\right) = \log x_{i_1}^2+\log x_{i_2}^2+\ldots\log x_{i_l}^2 \le l\cdot\log\left(\frac{x_{i_1}^2+x_{i_2}^2+\ldots x_{i_l}^2}{l}\right).$$

Since $x_{i_1}^2+x_{i_2}^2+\ldots x_{i_l}^2 \le \varepsilon$ for any $1 \le i_1 < i_2 < \cdots \le m$, it holds that

$$\begin{aligned}
\sum_{I_l}\exp\left\{l\cdot\log\left(\frac{x_{i_1}^2+x_{i_2}^2+\ldots x_{i_l}^2}{l}\right)\right\} &\le \sum_{I_l}\exp\left\{l\cdot\log\varepsilon l^{-1}\right\} \\
&= \sum_{I_l}O(\varepsilon^l)l^{-l}
\end{aligned}$$

for any l-term in the sum. Each successive summand is of growing order in ε, so we may truncate the entire expression at $l = 1$:

$$\bigoplus_{i=1}^{m}(\pm x_i^2) = \pm\,\|x\|_2^2 + O(\varepsilon^2).$$

□

Proof of Lemma 2. Begin the proof as per above. Borrowing notation from Equation 3,

$$\begin{aligned}
{}_q\|x\|^2 &= \bigoplus_{i=1}^{m}(-x_i^2) \\
&= \sum_{l=1}^{m}\sum_{I_l}(1-q)^{l-1}(-x_{i_1}^2)(-x_{i_2}^2)\cdots(-x_{i_l}^2) \\
&= \sum_{l=1}^{m}(-1)^l(1-q)^{l-1}\sum_{I_l}x_{i_1}^2 x_{i_2}^2\cdots x_{i_l}^2 \\
&= \sum_{l=1}^{m}(-1)^{2l-1}|1-q|^{l-1}\sum_{I_l}x_{i_1}^2 x_{i_2}^2\cdots x_{i_l}^2, \quad \text{since } q>1 \\
&= -\sum_{l=1}^{m}|1-q|^{l-1}\sum_{I_l}x_{i_1}^2 x_{i_2}^2\cdots x_{i_l}^2 \\
&\le -\sum_{i=1}^{m}x_i^2 \qquad \{\text{ keep the } (l=1)\text{-terms only}\} \\
&= -\|x\|_2^2.
\end{aligned}$$

□

References

[1] C. Hafemeister and R. Satija, "Normalization and variance stabilization of single-cell rna-seq data using regularized negative binomial regression," *Genome biology*, vol. 20, no. 1, pp. 1–15, 2019.

[2] R. S. Wedemann, A. R. Plastino, and C. Tsallis, "Curl forces and the nonlinear fokker-planck equation," *Phys. Rev. E*, vol. 94, p. 062 105, 6 Dec. 2016. DOI: `10.1103/PhysRevE.94.062105`. [Online]. Available: `https://link.aps.org/doi/10.1103/PhysRevE.94.062105`.

[3] L. Borland, "Microscopic dynamics of the nonlinear fokker-planck equation: A phenomenological model," *Physical Review E*, vol. 57, no. 6, pp. 6634–6642, 1998.

[4] A. Butler, P. Hoffman, P. Smibert, E. Papalexi, and R. Satija, "Integrating single-cell transcriptomic data across different conditions, technologies, and species," *Nature biotechnology*, vol. 36, no. 5, pp. 411–420, 2018.

[5] J. J. Dziak, D. L. Coffman, S. T. Lanza, R. Li, and L. S. Jermiin, "Sensitivity and specificity of information criteria," *Briefings in bioinformatics*, vol. 21, no. 2, pp. 553–565, 2020.

[6] D. Kotliar, A. Veres, M. A. Nagy, *et al.*, "Identifying gene expression programs of cell-type identity and cellular activity with single-cell rna-seq," *Elife*, vol. 8, 2019.

[7] P. D. Thomas, D. Ebert, A. Muruganujan, T. Mushayahama, L.-P. Albou, and H. Mi, "Panther: Making genome-scale phylogenetics accessible to all," *Protein Science*, vol. 31, no. 1, pp. 8–22, 2022. DOI: `https://doi.org/10.1002/pro.4218`. eprint: `https://onlinelibrary.wiley.com/doi/pdf/10.1002/pro.4218`. [Online]. Available: `https://onlinelibrary.wiley.com/doi/abs/10.1002/pro.4218`.

[8] H. Mi, A. Muruganujan, X. Huang, *et al.*, "Protocol update for large-scale genome and gene function analysis with the panther classification system (v. 14.0)," *Nature protocols*, vol. 14, no. 3, pp. 703–721, 2019.

[9] A. M. Newman, C. B. Steen, C. L. Liu, *et al.*, "Determining cell type abundance and expression from bulk tissues with digital cytometry," *Nature biotechnology*, vol. 37, no. 7, pp. 773–782, 2019.

[10] F. B. Knight, *Essentials of Brownian Motion and Diffusion.* American Mathematical Society, 1981.

[11] R. R. Coifman and S. Lafon, "Diffusion maps," *Applied and Computational Harmonic Analysis*, vol. 21, pp. 5–30, 2006.

[12] A. Haddad, D. Kushnir, and R. R. Coifman, "Texture separation via a reference set," *Applied and Computational Harmonic Analysis*, vol. 35, pp. 335–347, 2014.

[13] R. Jordan, D. Kinderlehrer, and F. Otto, *SIAM J. Math. Anal.*, vol. 29, no. 1, pp. 1–17, 2006.

[14] A. Ohara and T. Wada, "Information geometry of q-gaussian densities and behaviors of solutions to related diffusion equations," *Journal of Physics A*, vol. 43, p. 035 002, 2009.

[15] K. M. Carter, R. Raich, and A. O. Hero III, "On local intrinsic dimension estimation and its applications," *IEEE Transactions on Signal Processing*, vol. 58, no. 2, pp. 650–663, 2010.

[16] D. Arthur and S. Vassilvitskii, "K-means++: The advantages of careful seeding," Stanford, Tech. Rep., 2006.

[17] A. C. Gadotti, M. de Castro Deus, J. P. Telles, *et al.*, "Ifn-γ is an independent risk factor associated with mortality in patients with moderate and severe covid-19 infection," *Virus research*, vol. 289, p. 198 171, 2020.

[18] Z.-J. Hu, J. Xu, J.-M. Yin, *et al.*, "Lower circulating interferon-gamma is a risk factor for lung fibrosis in covid-19 patients," *Frontiers in immunology*, vol. 11, p. 585 647, 2020.

[19] L. Yu, Y. Cao, J. Y. Yang, and P. Yang, "Benchmarking clustering algorithms on estimating the number of cell types from single-cell rna-sequencing data," *Genome biology*, vol. 23, no. 1, pp. 1–21, 2022.

[20] X. Qiu, Q. Mao, Y. Tang, *et al.*, "Reversed graph embedding resolves complex single-cell trajectories," *Nature methods*, vol. 14, no. 10, pp. 979–982, 2017.

[21] F. A. Wolf, P. Angerer, and F. J. Theis, "Scanpy: Large-scale single-cell gene expression data analysis," *Genome biology*, vol. 19, no. 1, pp. 1–5, 2018.

[22] V. Y. Kiselev, K. Kirschner, M. T. Schaub, *et al.*, "Sc3: Consensus clustering of single-cell rna-seq data," *Nature methods*, vol. 14, no. 5, pp. 483–486, 2017.

[23] G.-J. Huizing, G. Peyré, and L. Cantini, "Optimal transport improves cell–cell similarity inference in single-cell omics data," *Bioinformatics*, vol. 38, no. 8, pp. 2169–2177, 2022.

[24] A. Duò, M. D. Robinson, and C. Soneson, "A systematic performance evaluation of clustering methods for single-cell rna-seq data," *F1000Research*, vol. 7, p. 1141, 2018. DOI: `10.12688/f1000research.15666.3`.

[25] R. Bellazzi, A. Codegoni, S. Gualandi, G. Nicora, and E. Vercesi, "The gene mover's distance: Single-cell similarity via optimal transport," *arXiv preprint arXiv:2102.01218*, 2021.

[26] K. Pham, K. Le, N. Ho, T. Pham, and H. Bui, "On unbalanced optimal transport: An analysis of sinkhorn algorithm," in *International Conference on Machine Learning*, PMLR, 2020, pp. 7673–7682.

[27] C. Xu and Z. Su, "Identification of cell types from single-cell transcriptomes using a novel clustering method," *Bioinformatics*, vol. 31, no. 12, pp. 1974–1980, Feb. 2015, ISSN: 1367-4803. DOI: `10.1093/bioinformatics/btv088`. eprint: `https://academic.oup.com/bioinformatics/article-pdf/31/12/1974/17100675/btv088.pdf`. [Online]. Available: `https://doi.org/10.1093/bioinformatics/btv088`.

[28] J. H. Levine, E. F. Simonds, S. C. Bendall, *et al.*, "Data-driven phenotypic dissection of aml reveals progenitor-like cells that correlate with prognosis," *Cell*, vol. 162, no. 1, pp. 184–197, 2015.

[29] Y.-H. Uen, C.-L. Fang, Y.-C. Hseu, *et al.*, "Vav3 oncogene expression in colorectal cancer: Clinical aspects and functional characterization," *Scientific reports*, vol. 5, no. 1, pp. 1–8, 2015.

[30] R. Kuang, A. Jahangiri, S. Mascharak, *et al.*, "Glut3 upregulation promotes metabolic reprogramming associated with antiangiogenic therapy resistance," *JCI insight*, vol. 2, no. 2, 2017.

[31] M. Tsukioka, Y. Matsumoto, M. Noriyuki, *et al.*, "Expression of glucose transporters in epithelial ovarian carcinoma: Correlation with clinical characteristics and tumor angiogenesis," *Oncology reports*, vol. 18, no. 2, pp. 361–367, 2007.

[32] A. Semaan, A. R. Munkarah, H. Arabi, *et al.*, "Expression of glut-1 in epithelial ovarian carcinoma: Correlation with tumor cell proliferation, angiogenesis, survival and ability to predict optimal cytoreduction," *Gynecologic oncology*, vol. 121, no. 1, pp. 181–186, 2011.

[33] X. Yang, J. Zhong, Q. Zhang, *et al.*, "Advances and insights of apc-asef inhibitors for metastatic colorectal cancer therapy," *Frontiers in Molecular Biosciences*, vol. 8, p. 662 579, 2021.

[34] Y. Kawasaki, S. Tsuji, K. Muroya, *et al.*, "The adenomatous polyposis coli-associated exchange factors asef and asef2 are required for adenoma formation in apcmin/+ mice," *EMBO reports*, vol. 10, no. 12, pp. 1355–1362, 2009.

[35] Y. Kawasaki, T. Jigami, S. Furukawa, *et al.*, "The adenomatous polyposis coli-associated guanine nucleotide exchange factor asef is involved in angiogenesis," *Journal of Biological Chemistry*, vol. 285, no. 2, pp. 1199–1207, 2010.

[36] H. Chen, X. Sun, W. Ge, Y. Qian, R. Bai, and S. Zheng, "A seven-gene signature predicts overall survival of patients with colorectal cancer," *Oncotarget*, vol. 8, no. 56, p. 95 054, 2017.

[37] E. M. Meškytė, S. Keskas, and Y. Ciribilli, "Myc as a multifaceted regulator of tumor microenvironment leading to metastasis," *International journal of molecular sciences*, vol. 21, no. 20, p. 7710, 2020.

[38] K. Sakamoto, S. Maeda, Y. Hikiba, *et al.*, "Constitutive nf-κb activation in colorectal carcinoma plays a key role in angiogenesis, promoting tumor growth," *Clinical Cancer Research*, vol. 15, no. 7, pp. 2248–2258, 2009.

[39] M. Rodriguez, I. Egana, F. Lopitz-Otsoa, *et al.*, "The ring ubiquitin e3 rnf114 interacts with a20 and modulates nf-κb activity and t-cell activation," *Cell death & disease*, vol. 5, no. 8, e1399–e1399, 2014.

[40] L. Zheng, N. You, X. Huang, *et al.*, "Commd7 regulates nf-κb signaling pathway in hepatocellular carcinoma stem-like cells," *Molecular Therapy-Oncolytics*, vol. 12, pp. 112–123, 2019.

[41] N. Cao, Y. Yu, H. Zhu, *et al.*, "Setdb1 promotes the progression of colorectal cancer via epigenetically silencing p21 expression," *Cell Death & Disease*, vol. 11, no. 5, p. 351, 2020.

[42] J. M. Balko and E. P. Black, "A gene expression predictor of response to egfr-targeted therapy stratifies progression-free survival to cetuximab in kras wild-type metastatic colorectal cancer," *BMC cancer*, vol. 9, pp. 1–10, 2009.

[43] N. M. Kofler, C. J. Shawber, T. Kangsamaksin, H. O. Reed, J. Galatioto, and J. Kitajewski, "Notch signaling in developmental and tumor angiogenesis," *Genes & cancer*, vol. 2, no. 12, pp. 1106–1116, 2011.

[44] B. Parang, A. M. Bradley, M. K. Mittal, *et al.*, "Myeloid translocation genes differentially regulate colorectal cancer programs," *Oncogene*, vol. 35, no. 49, pp. 6341–6349, 2016.

[45] R. Benelli, D. Costa, L. Mastracci, *et al.*, "Aspartate-β-hydroxylase: A promising target to limit the local invasiveness of colorectal cancer," *Cancers*, vol. 12, no. 4, p. 971, 2020.

[46] R. Cruz-Duarte, C. Rebelo de Almeida, M. Negrão, *et al.*, "Predictive and therapeutic implications of a novel plcγ1/shp2-driven mechanism of cetuximab resistance in metastatic colorectal cancerplcγ1: Clinical implications for cetuximab therapy," *Clinical Cancer Research*, OF1–OF14, 2022.

[47] M. Zhang, H.-z. Wang, H.-o. Li, *et al.*, "Identification of pigu as the hub gene associated with kras mutation in colorectal cancer by coexpression analysis," *DNA and Cell Biology*, vol. 39, no. 9, pp. 1639–1648, 2020.

[48] M. Li, F. Keshavarz-Rahaghi, G. Ladua, *et al.*, "Characterizing the kras g12c mutation in metastatic colorectal cancer: A population-based cohort and assessment of expression differences in the cancer genome atlas," *Therapeutic Advances in Medical Oncology*, vol. 14, p. 17 588 359 221 097 940, 2022.

[49] F. Conciatori, L. Ciuffreda, C. Bazzichetto, *et al.*, "Mtor cross-talk in cancer and potential for combination therapy," *Cancers*, vol. 10, no. 1, p. 23, 2018.

[50] H. Lotfimehr, N. Mardi, S. Narimani, *et al.*, "Mtor signalling pathway in stem cell bioactivities and angiogenesis potential," *Cell Proliferation*, e13499, 2023.

[51] P. Xu, G. Du, H. Guan, W. Xiao, L. Sun, and H. Yang, "A role of tti1 in the colorectal cancer by promoting proliferation," *Translational Cancer Research*, vol. 10, no. 3, p. 1378, 2021.

[52] Z. Guo, X. Zhang, H. Zhu, *et al.*, "Telo2 induced progression of colorectal cancer by binding with rictor through mtorc2," *Oncology reports*, vol. 45, no. 2, pp. 523–534, 2021.

[53] Y.-W. Kim and T. V. Byzova, "Oxidative stress in angiogenesis and vascular disease," *Blood, the Journal of the American Society of Hematology*, vol. 123, no. 5, pp. 625–631, 2014.

[54] N. Traverso, R. Ricciarelli, M. Nitti, *et al.*, "Role of glutathione in cancer progression and chemoresistance," *Oxidative medicine and cellular longevity*, vol. 2013, 2013.

[55] A. D. Kim, R. Zhang, X. Han, *et al.*, "Involvement of glutathione and glutathione metabolizing enzymes in human colorectal cancer cell lines and tissues," *Molecular medicine reports*, vol. 12, no. 3, pp. 4314–4319, 2015.

[56] Q. Dang, Z. Liu, S. Hu, *et al.*, "Derivation and clinical validation of a redox-driven prognostic signature for colorectal cancer," *Frontiers in Oncology*, vol. 11, p. 743 703, 2021.

[57] T. Ismail, Y. Kim, H. Lee, D.-S. Lee, and H.-S. Lee, "Interplay between mitochondrial peroxiredoxins and ros in cancer development and progression," *International journal of molecular sciences*, vol. 20, no. 18, p. 4407, 2019.

[58] H.-M. Ahn, J.-W. Yoo, S. Lee, H. J. Lee, H.-S. Lee, and D.-S. Lee, "Peroxiredoxin 5 promotes the epithelial-mesenchymal transition in colon cancer," *Biochemical and Biophysical Research Communications*, vol. 487, no. 3, pp. 580–586, 2017.

[59] A. Fantozzi, D. C. Gruber, L. Pisarsky, *et al.*, "Vegf-mediated angiogenesis links emt-induced cancer stemness to tumor initiationemt, stemness, and angiogenesis," *Cancer research*, vol. 74, no. 5, pp. 1566–1575, 2014.

[60] Y. Sun, B. Liu, Y. Chen, Y. Xing, and Y. Zhang, "Multi-omics prognostic signatures based on lipid metabolism for colorectal cancer," *Frontiers in Cell and Developmental Biology*, vol. 9, p. 3705, 2022.

[61] E. Gharib, P. Nasrinasrabadi, and M. R. Zali, "Development and validation of a lipogenic genes panel for diagnosis and recurrence of colorectal cancer," *PLoS One*, vol. 15, no. 3, e0229864, 2020.

[62] P. Alves, N. Lévy, B. J. Stevenson, *et al.*, "Identification of tumor-associated antigens by large-scale analysis of genes expressed in human colorectal cancer," *Cancer immunity*, vol. 8, no. 1, 2008.

[63] Z. Zhang, A. Banerjee, K. Baksi, and D. K. Banerjee, "Mannosylphosphodolichol synthase overexpression supports angiogenesis," *Biocatalysis and biotransformation*, vol. 28, no. 1, pp. 90–98, 2010.

[64] K. Baksi, Z. Zhang, A. Banerjee, *et al.*, "Silencing mannosylphospho dolichol synthase with shrna impacts differentiation of capillary endothelial cells," *The FASEB Journal*, vol. 30, pp. 844–1, 2016.

[65] S. Stintzing, B. Ivanova, I. Ricard, *et al.*, "Amphiregulin (areg) and epiregulin (ereg) gene expression as predictor for overall survival (os) in oxaliplatin/fluoropyrimidine plus bevacizumab treated mcrc patients—analysis of the phase iii aio krk-0207 trial," *Frontiers in Oncology*, vol. 8, p. 474, 2018.

[66] H. Hashida and R. J. Coffey, "Significance of a calcium-binding protein s100a14 expression in colon cancer progression," *Journal of Gastrointestinal Oncology*, vol. 13, no. 1, p. 149, 2022.

[67] D. Massi, M. Landriscina, A. Piscazzi, *et al.*, "S100a13 is a new angiogenic marker in human melanoma," *Modern Pathology*, vol. 23, no. 6, pp. 804–813, 2010.

[68] Y. Wang, L. Hu, Y. Zheng, and L. Guo, "Hmga1 in cancer: Cancer classification by location," *Journal of Cellular and Molecular Medicine*, vol. 23, no. 4, pp. 2293–2302, 2019.

[69] R. Zanin, S. Pegoraro, G. Ros, *et al.*, "Hmga1 promotes breast cancer angiogenesis supporting the stability, nuclear localization and transcriptional activity of foxm1," *Journal of Experimental & Clinical Cancer Research*, vol. 38, pp. 1–23, 2019.

[70] L. Resar, L. Chia, and L. Xian, "Lessons from the crypt: Hmga1—amping up wnt for stem cells and tumor progression," *Cancer research*, vol. 78, no. 8, pp. 1890–1897, 2018.

[71] M. D. Williams, X. Zhang, A. S. Belton, *et al.*, "Hmga1 drives metabolic reprogramming of intestinal epithelium during hyperproliferation, polyposis, and colorectal carcinogenesis," *Journal of proteome research*, vol. 14, no. 3, pp. 1420–1431, 2015.

[72] J. Chi, H. Zhang, J. Hu, *et al.*, "Agr3 promotes the stemness of colorectal cancer via modulating wnt/β-catenin signalling," *Cellular signalling*, vol. 65, p. 109 419, 2020.

[73] M. K. Skytthe, J. H. Graversen, and S. K. Moestrup, "Targeting of cd163+ macrophages in inflammatory and malignant diseases," *International journal of molecular sciences*, vol. 21, no. 15, p. 5497, 2020.

[74] T. Xue, K. Yan, Y. Cai, *et al.*, "Prognostic significance of cd163+ tumor-associated macrophages in colorectal cancer," *World journal of surgical oncology*, vol. 19, no. 1, pp. 1–7, 2021.

[75] D. Krijgsman, N. L. De Vries, M. N. Andersen, *et al.*, "Cd163 as a biomarker in colorectal cancer: The expression on circulating monocytes and tumor-associated macrophages, and the soluble form in the blood," *International journal of molecular sciences*, vol. 21, no. 16, p. 5925, 2020.

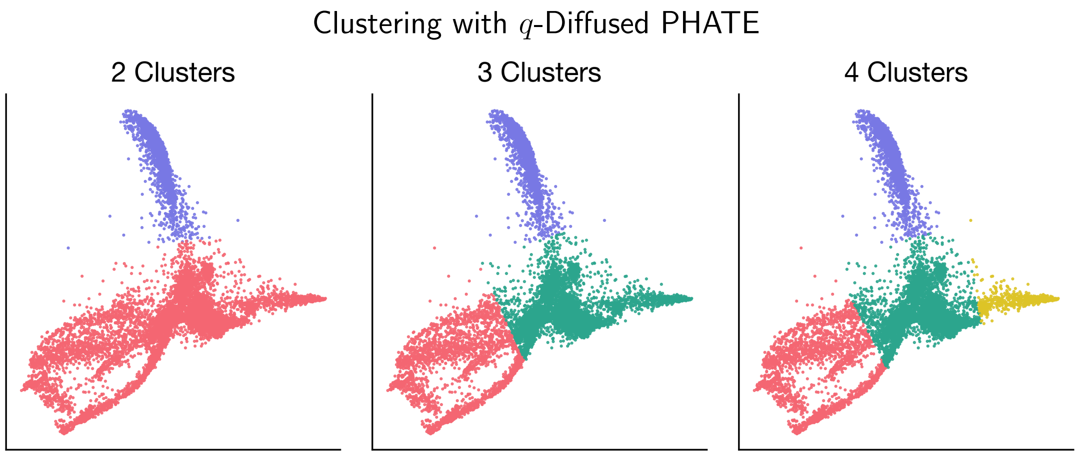

(a) q-Diffused embedding colored by k-means clusterings [16] for $k \in \{2, 3, 4\}$. The three versions serve to validate the stability of the two major branches (blue, red) and one minor branch (yellow).

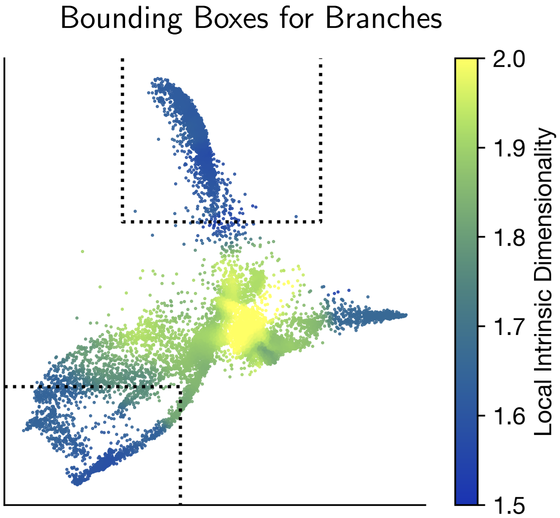

(b) q-Diffused embedding colored by the same LID estimates as in Figure 3a, here annotated with the precise bounding boxes that delineated the cells of the two major branches for downstream analysis.

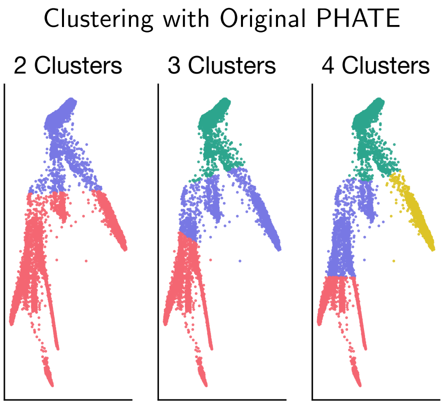

(c) Original embedding colored by their k-means clusterings, as with (a). Evidently, the existence of major branches is less stable: observe how red and blue sections continuously shift.

Supplementary Figure 3. Justification for the branching classifications in Figure 3 for mCRC. We synthesized the LID and the stable k-means clusterings to draw bounding boxes on the q-diffused PHATE embedding.
