# Supplementary material for: q-Diffusion leverages the full dimensionality of gene coexpression in single-cell transcriptomics
Source: Commun Biol. 2024 Apr 2;7:400. doi: 10.1038/s42003-024-06104-w (PMC11255321; doi:10.1038/s42003-024-06104-w)
Supplement: Supplementary file 2 — Description of Supplementary Materials [file 42003_2024_6104_MOESM2_ESM.docx]

**Description of Additional Supplementary Files**

**File name:** Supplementary Data 1

**Description:** Collection of tables containing the numerical results for the charts
