# Supplementary material for: q-Diffusion leverages the full dimensionality of gene coexpression in single-cell transcriptomics
Source: Commun Biol. 2024 Apr 2;7:400. doi: 10.1038/s42003-024-06104-w (PMC11255321; doi:10.1038/s42003-024-06104-w)
Supplement: Supplementary file 3 — Reporting Summary [file 42003_2024_6104_MOESM3_ESM.pdf]

nature portfolio

Corresponding author(s): Myrl G. Marmarelis

Last updated by author(s): Jan 21, 2024

Reporting Summary

Nature Portfolio wishes to improve the reproducibility of the work that we publish. This form provides structure for consistency and transparency in reporting. For further information on Nature Portfolio policies, see our Editorial Policies and the Editorial Policy Checklist.

Statistics

For all statistical analyses, confirm that the following items are present in the figure legend, table legend, main text, or Methods section.

n/a	Confirmed	
☐	☒	The exact sample size (*n*) for each experimental group/condition, given as a discrete number and unit of measurement
☐	☒	A statement on whether measurements were taken from distinct samples or whether the same sample was measured repeatedly
☐	☒	The statistical test(s) used AND whether they are one- or two-sided<br>*Only common tests should be described solely by name; describe more complex techniques in the Methods section.*
☐	☒	A description of all covariates tested
☐	☒	A description of any assumptions or corrections, such as tests of normality and adjustment for multiple comparisons
☐	☒	A full description of the statistical parameters including central tendency (e.g. means) or other basic estimates (e.g. regression coefficient) AND variation (e.g. standard deviation) or associated estimates of uncertainty (e.g. confidence intervals)
☐	☒	For null hypothesis testing, the test statistic (e.g. F, t, r) with confidence intervals, effect sizes, degrees of freedom and P value noted<br>*Give P values as exact values whenever suitable.*
☐	☒	For Bayesian analysis, information on the choice of priors and Markov chain Monte Carlo settings
☐	☒	For hierarchical and complex designs, identification of the appropriate level for tests and full reporting of outcomes
☐	☒	Estimates of effect sizes (e.g. Cohen's *d*, Pearson's *r*), indicating how they were calculated

Our web collection on statistics for biologists contains articles on many of the points above.

Software and code

Policy information about availability of computer code

Data collection	We only analyzed previously collected data in this study.
Data analysis	Open-source libraries for the Julia and Python programming languages were used in conjunction with custom source code, which has all been deposited on github.com/marmarelis/QDiffusion.jl .

For manuscripts utilizing custom algorithms or software that are central to the research but not yet described in published literature, software must be made available to editors and reviewers. We strongly encourage code deposition in a community repository (e.g. GitHub). See the Nature Portfolio guidelines for submitting code & software for further information.

Data

Policy information about availability of data

All manuscripts must include a data availability statement. This statement should provide the following information, where applicable:

- Accession codes, unique identifiers, or web links for publicly available datasets
- A description of any restrictions on data availability
- For clinical datasets or third party data, please ensure that the statement adheres to our policy

All datasets considered in this study have been previously reported in the literature. Accession codes or links are provided for all datasets besides the clinical trial: the Human Colon Cancer Atlas can be accessed at GEO: GSE178341; the PBMC benchmark at GEO: GSE96583; the Tabula Sapiens benchmark at https://

doi.org/10.6084/m9.figshare.14267219.v5 ; the human MERFISH sample at https://doi.org/10.5061/dryad.x3ffbg7mw . For the CALGB/SWOG 80405 clinical trial, a summary of clinical and genomic data will be made available upon reasonable request.

Research involving human participants, their data, or biological material

Policy information about studies with human participants or human data. See also policy information about sex, gender (identity/presentation), and sexual orientation and race, ethnicity and racism.

Reporting on sex and gender	In the clinical-trial case study, we used sex as a covariate in the multivariate analysis, in accordance with the protocol of that clinical trial. We did not consider biological sex in the other two case studies because their scope was to benchmark a method, not to make biological discoveries.
Reporting on race, ethnicity, or other socially relevant groupings	We did not consider groupings like race or ethnicity in our analysis, nor did we consider socioeconomic attributes. The covariates for the clinical-trial case study included bulk RNA sequencing, sex, mutations, and other tumor characteristics.
Population characteristics	Our study is a retrospective analysis on previously reported data.
Recruitment	As mentioned, our study is a retrospective analysis on previously reported data. For this information regarding the clinical trial, please view details here: https://clinicaltrials.gov/study/NCT00265850 .
Ethics oversight	This study was conducted in accordance with guidelines of the Declaration of Helsinki, Belmont report, Good Clinical Practice, REMARK, and U.S. Common Rule. Patients from the CALGB/SWOG 80405 trial provided written informed consent granting permission for molecular research to be performed on submitted tumor samples prior to study enrollment. The study protocol was approved by the IRB of each participating institution.<br>All analyses for the clinical-trial case study were performed using retrospective, de-identified clinical data.

Note that full information on the approval of the study protocol must also be provided in the manuscript.

Field-specific reporting

Please select the one below that is the best fit for your research. If you are not sure, read the appropriate sections before making your selection.

☒ Life sciences ☐ Behavioural & social sciences ☐ Ecological, evolutionary & environmental sciences

For a reference copy of the document with all sections, see nature.com/documents/nr-reporting-summary-flat.pdf

Life sciences study design

All studies must disclose on these points even when the disclosure is negative.

Sample size	We limited our analysis to previously collected datasets, including atlases and clinical trials.
Data exclusions	Data points were not excluded beyond any steps taken in the original studies that collected the datasets. For scRNAseq, we followed standard preprocessing procedures that excluded cells with low-quality reads.
Replication	We do not believe this is applicable because we did not collect any new data. For the reproducibility of our analyses, we have released full source code and listed the random seeds we used. We also reran the algorithms on bootstrap-resampled data whenever feasible and pertinent.
Randomization	We do not believe this is applicable because we did not collect any new data. Details are provided in the various referenced studies that originally collected the datasets we analyzed.
Blinding	All data were already de-identified.

Reporting for specific materials, systems and methods

We require information from authors about some types of materials, experimental systems and methods used in many studies. Here, indicate whether each material, system or method listed is relevant to your study. If you are not sure if a list item applies to your research, read the appropriate section before selecting a response.

Materials & experimental systems

n/a	Involved in the study
☒	☐ Antibodies
☒	☐ Eukaryotic cell lines
☒	☐ Palaeontology and archaeology
☒	☐ Animals and other organisms
☒	☐ Clinical data
☒	☐ Dual use research of concern
☒	☐ Plants

Methods

n/a	Involved in the study
☒	☐ ChIP-seq
☒	☐ Flow cytometry
☒	☐ MRI-based neuroimaging
